# Mutated in colorectal cancer (MCC) is a novel oncogene in B lymphocytes

**DOI:** 10.1186/s13045-014-0056-6

**Published:** 2014-09-09

**Authors:** Shanique KE Edwards, Jacqueline Baron, Carissa R Moore, Yan Liu, David H Perlman, Ronald P Hart, Ping Xie

**Affiliations:** Department of Cell Biology and Neuroscience, Piscataway, NJ 08854 USA; Graduate Program in Molecular Biosciences, Piscataway, NJ 08854 USA; W.M. Keck Center for Collaborative Neuroscience, Rutgers University, Piscataway, NJ 08854 USA; Princeton Collaborative Proteomics & Mass Spectrometry Center, Lewis-Sigler Institute of Integrative Genomics, Princeton University, Princeton, NJ 08544 USA; Rutgers Cancer Institute of New Jersey, New Brunswick, USA; Department of Cell Biology and Neuroscience, Rutgers University, 604 Allison Road, Nelson Labs Room B336, Piscataway, NJ 08854 USA

**Keywords:** MCC, TRAF3, B lymphoma, Multiple myeloma, PARP1, PHB2

## Abstract

**Background:**

Identification of novel genetic risk factors is imperative for a better understanding of B lymphomagenesis and for the development of novel therapeutic strategies. TRAF3, a critical regulator of B cell survival, was recently recognized as a tumor suppressor gene in B lymphocytes. The present study aimed to identify novel oncogenes involved in malignant transformation of TRAF3-deficient B cells.

**Methods:**

We used microarray analysis to identify genes differentially expressed in TRAF3^−/−^ mouse splenic B lymphomas. We employed lentiviral vector-mediated knockdown or overexpression to manipulate gene expression in human multiple myeloma (MM) cell lines. We analyzed cell apoptosis and proliferation using flow cytometry, and performed biochemical studies to investigate signaling mechanisms. To delineate protein-protein interactions, we applied affinity purification followed by mass spectrometry-based sequencing.

**Results:**

We identified *mutated in colorectal cancer* (*MCC*) as a gene strikingly up-regulated in TRAF3-deficient mouse B lymphomas and human MM cell lines. Aberrant up-regulation of *MCC* also occurs in a variety of primary human B cell malignancies, including non-Hodgkin lymphoma (NHL) and MM. In contrast, *MCC* expression was not detected in normal or premalignant TRAF3^−/−^ B cells even after treatment with B cell stimuli, suggesting that aberrant up-regulation of *MCC* is specifically associated with malignant transformation of B cells. In elucidating the functional roles of MCC in malignant B cells, we found that lentiviral shRNA vector-mediated knockdown of MCC induced apoptosis and inhibited proliferation in human MM cells. Experiments of knockdown and overexpression of MCC allowed us to identify several downstream targets of MCC in human MM cells, including phospho-ERK, c-Myc, p27, cyclin B1, Mcl-1, caspases 8 and 3. Furthermore, we identified 365 proteins (including 326 novel MCC-interactors) in the MCC interactome, among which PARP1 and PHB2 were two hubs of MCC signaling pathways in human MM cells.

**Conclusions:**

Our results indicate that in sharp contrast to its tumor suppressive role in colorectal cancer, MCC functions as an oncogene in B cells. Our findings suggest that MCC may serve as a diagnostic marker and therapeutic target in B cell malignancies, including NHL and MM.

**Electronic supplementary material:**

The online version of this article (doi:10.1186/s13045-014-0056-6) contains supplementary material, which is available to authorized users.

## Background

B cell neoplasms account for over 90% of lymphoid tumors worldwide, and comprise >50% of blood cancers. Despite recent advances in treatment, many types of human B cell lymphomas remain incurable, highlighting a clear need for new preventative and therapeutic strategies [1,2]. Increasing evidence indicates the importance of genetic determinants in B lymphomagenesis [3–5]. Identification and validation of new genetic risk factors are imperative for a better understanding of B cell malignant transformation and for the development of new therapeutic strategies. Recent studies from our laboratory and others have identified TRAF3, a critical determinant of B cell survival, as a tumor suppressor gene in B lymphocytes. TRAF3 is a member of the tumor necrosis factor receptor (TNF-R)-associated factor (TRAF) family (TRAF1-6) of cytoplasmic adaptor proteins [6]. All TRAF proteins have the distinctive feature of a C-terminal TRAF domain, which mediates the interaction with TRAF-binding motifs of receptors of the TNF-R superfamily [6,7]. Homozygous deletions and inactivating mutations of the TRAF3 gene have been identified in non-Hodgkin lymphoma (NHL)**,** including splenic marginal zone lymphoma (MZL), B cell chronic lymphocytic leukemia (B-CLL) and mantle cell lymphoma (MCL), as well as multiple myeloma (MM) and Waldenström’s macroglobulinemia (WM) [8–11].

By generating and characterizing a mouse model that has the *Traf3* gene specifically deleted in B lymphocytes (B-TRAF3^−/−^ mice), we recently reported that TRAF3 deletion leads to spontaneous development of MZL and B1 lymphoma in mice [12,13]. Interestingly however, B lymphoma development in B-TRAF3^−/−^ mice exhibits a long latency (approximately 9 months), indicating that TRAF3 inactivation and its aberrant signaling pathways are not sufficient to induce B lymphomagenesis and that additional oncogenic pathways are necessary for B lymphoma development. Although TRAF3 deletions or mutations exist in human patients with NHL and MM, it is not known whether TRAF3 inactivation is the primary or secondary oncogenic mutation in human samples. Thus, B-TRAF3^−/−^ mice offer the unique advantage to identify secondary oncogenic pathways that drive B lymphomagenesis in the context of TRAF3 inactivation. To identify such secondary oncogenic alterations that mediate the malignant transformation of TRAF3^−/−^ B cells, we performed a transcriptome microarray analysis using TRAF3^−/−^ mouse splenic B lymphomas. Surprisingly, our microarray analysis identified *mutated in colorectal cancer* (*MCC*), a tumor suppressor gene of colorectal cancer, as a strikingly up-regulated gene in B lymphomas spontaneously developed in different individual B-TRAF3^−/−^ mice.

The *MCC* gene was discovered in 1991 through its linkage to the region showing loss of heterozygosity (LOH) in familial adenomatous polyposis (FAP) [14–17]. Subsequent studies revealed that the *adenomatous polyposis coli* (*APC*) gene and not *MCC* is responsible for FAP. The APC gene is mutated somatically in 60–80% of sporadic colorectal cancers (CRCs), whereas somatic mutation of MCC is relatively rare, 3–7%, in sporadic CRCs [14–18]. However, it was subsequently reported that the MCC gene is silenced through promoter methylation in approximately 50% of primary sporadic CRCs and 80% of serrated polyps, suggesting that the silencing of MCC is important in early colon carcinogenesis via the serrated neoplasia pathway [19–22]. Furthermore, loss-of-function mutations, LOH, or decreased expression of the *MCC* gene are also detected in a number of other human cancers, including lung cancer [17,23], gastric carcinoma [24], esophageal cancer [25], and hepatocellular carcinoma [26,27]. In addition, an SNP of the MCC gene (rs11283943) is significantly associated with increased risk of breast cancer [28]. Although an inactivating *MCC* mutation in mice alone failed to induce any evident CRCs, the homozygous mice displayed a slightly higher proliferation rate of the epithelial crypt cells [29,30]. Interestingly, an unbiased genetic screening of a mouse model of CRC implicated *MCC* mutation as a key event in colorectal carcinogenesis [18]. Consistent with the genetic evidence, functional studies revealed that MCC blocks cell cycle progression in NIH3T3 fibroblasts and CRCs [31,32], inhibits cell proliferation and migration in CRCs [20,32–34], and is required for DNA damage response in CRCs [35]. MCC appears to specifically target and negatively regulate the oncogenic NF-κB and β-catenin pathways in CRCs and hepatocellular carcinoma [20,27,32,36]. Mutation studies have revealed that the N-terminal domain (130–278 aa) of MCC is required for repressing the Wnt/β-catenin signaling pathway [20] and that the PDZ-binding motif at the extreme C-terminus of MCC mediates its interaction with Scrib-Myosin IIB to regulate cytoskeletal reorganization and cell migration in CRCs [34]. Collectively, the above genetic and functional evidence indicates that *MCC* functions as a tumor suppressor gene in CRCs by inhibiting cell cycle progression and migration, and by promoting DNA damage-induced cell cycle arrest in colorectal epithelial cells.

It has been shown that during development, MCC is expressed in diverse tissues derived from all three embryonic germ layers, including the developing gut and central nervous system [29]. In adults, MCC is expressed in the surface epithelium of the colon and villi of the small intestines as well as other tissues, including the cerebellar cortex, kidney, pancreas, and liver [31,37]. However, expression of MCC was not reported in lymphocytes, and the function of MCC in lymphocytes or lymphomas has not been explored. In the present study, we aimed to address this gap in knowledge.

Our unexpected finding that *MCC* was strikingly up-regulated in TRAF3^−/−^ mouse B lymphomas prompted us to further examine *MCC* expression in human B cell neoplasms. Our results demonstrated high levels of aberrant *MCC* expression in 6 human patient-derived MM cell lines with TRAF3 deletions or relevant mutations. We also surveyed the public gene expression database of microarray data of human cancers (http://www.oncomine.org), and learned that *MCC* was also aberrantly and significantly elevated in a variety of primary human B cell malignancies. These include primary effusion lymphoma (PEL), centroblastic lymphoma (CBL), diffuse large B-cell lymphoma (DLBCL), Burkitt’s lymphoma (BL), and MM [38–41]. However, expression of the transcript and protein of MCC was not detected in wild type B cells or premalignant TRAF3^−/−^ B cells, even after treatment with a variety of B cell stimuli, including CD40, BCR, LPS, and CpG. These results suggest that aberrant MCC expression is associated specifically with B cell tumorigenesis. We next investigated the functions of MCC in malignant B cell survival and proliferation, and found that lentiviral shRNA vector-mediated knockdown of MCC induced apoptosis and inhibited proliferation in human patient-derived MM cell lines. Furthermore, we identified MCC signaling pathways and interacting partners in malignant B cells. Our results thus provide novel mechanistic insights into how MCC promotes malignant B cell survival and proliferation. Taken together, our findings indicate that in sharp contrast to its tumor suppressive function in CRCs and other human carcinomas, MCC is oncogenic in B lymphocytes.

## Results

### Transcriptome microarray analysis of TRAF3^-/-^ mouse B lymphomas

**Figure 1:**
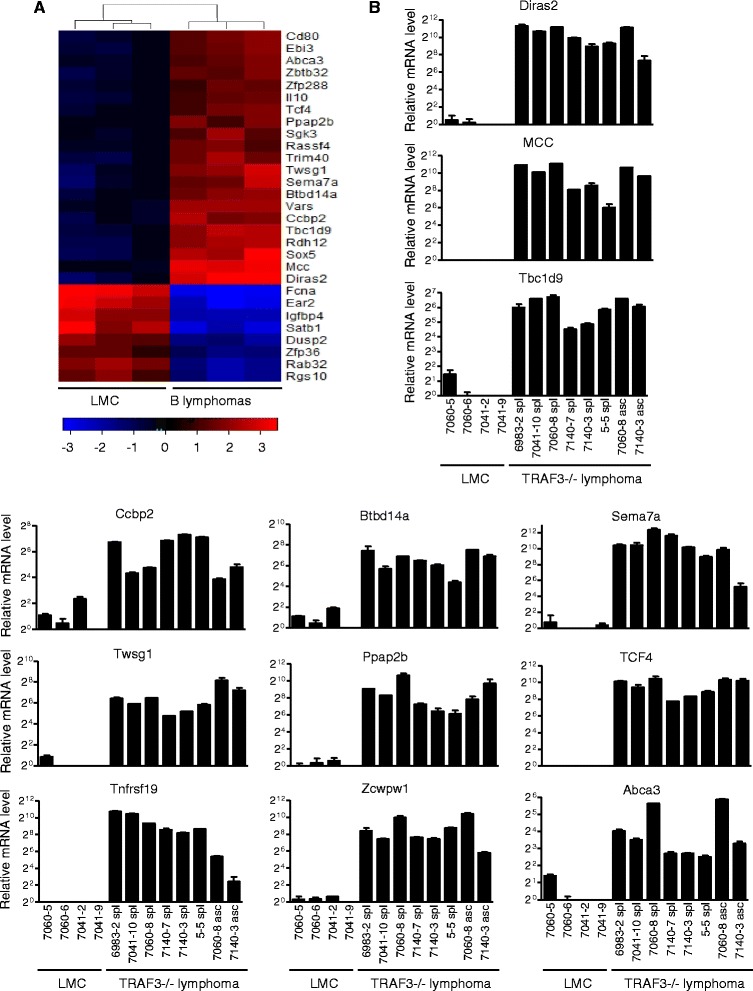
**Representative genes differentially expressed in TRAF3** ^**−/−**^ **mouse B lymphomas identified by the transcriptome microarray analysis. (A)** Heatmap of representative microarray data**.** The mRNA expression profiles of splenocytes from 3 pairs of LMC and tumor-bearing B-TRAF3^−/−^ mice were analyzed by microarray analysis. cRNA was hybridized to the Illumina Sentrix MouseRef-8 24 K Array (Illumina). Genes shown in the heatmap are selected from 160 up-regulated and 244 down-regulated genes: red indicates overexpression, blue indicates underexpression, and black indicates median expression. Values under the color key indicates Log2 (fold of change). Dendrogram on top of the heatmap shows the relatedness between samples as determined by hierarchical clustering. **(B)** Verification of transcript up-regulation of genes identified by the microarray analysis using quantitative real time PCR. Total cellular RNA was prepared from splenocytes of LMC mice, or splenic B lymphomas (spl) and ascites (asc) of diseased B-TRAF3^−/−^ mice, and cDNA was synthesized by reverse transcription. Real time PCR was performed using TaqMan primers and probes (FAM-labeled) specific for mouse *Diras2*, *MCC*, *Tbc1d9*, *Ccbp2*, *Btbd14a*, *Sema7a*, *Twsg1*, *Ppap2b*, *TCF4*, *Tnfrsf19*, *Zcwpw1*, and *Abca3*. Each reaction also included the probe (VIC-labeled) and primers for mouse β-actin mRNA, which served as endogenous control. Relative mRNA expression levels of each gene were analyzed using the Sequencing Detection Software (Applied Biosystems) and the comparative Ct (ΔΔCt) method. Graphs depict the results of two experiments with duplicate reactions in each experiment (mean ± S.D.), and mouse ID of each sample is indicated in the graphs.

TRAF3^−/−^ B cells purified from young B-TRAF3^−/−^ mice exhibit prolonged survival but do not proliferate autonomously [12], and therefore are premalignant B cells. Consistent with this, no B lymphoma development was observed in B-TRAF3^−/−^ mice younger than 9 months old [13]. The long latency of B lymphoma development observed in B-TRAF3^−/−^ mice suggests that TRAF3 inactivation and its downstream signaling pathways are not sufficient and that additional oncogenic alterations are required to induce B lymphomagenesis. To identify such secondary oncogenic alterations, to provide new insights into the molecular mechanisms of B cell malignant transformation, and to discover new therapeutic targets for the treatment of B cell malignancies, we performed global gene expression profiling of TRAF3^−/−^ mouse B lymphomas by transcriptome microarray analysis. We used RNA samples of 3 representative TRAF3^−/−^ splenic B lymphomas (mouse ID: 6983–2, 7041–10, and 7060–8), in which B lymphomas are >70% of B cells, as assessed by FACS analysis of B cell populations and Southern blot analysis of IgH gene rearrangements [13]. Results of the microarray analysis have identified 160 up-regulated genes and 244 down-regulated genes in TRAF3^−/−^ B lymphomas as compared to LMC spleens (cut-off fold of changes: 2-fold up or down, *p* < 0.05; Additional file 1: Table S1) (NCBI GEO accession number: GSE48818). Selective examples of these identified genes are shown in the heatmap (Figure 1A). Functional clustering and pathway analyses by Ingenuity (http://www.ingenuity.com) revealed that genes differentially expressed in TRAF3^−/−^ B lymphomas include transcription factors, cell surface receptors, enzymes, cell cycle regulators, protein translation regulators, lipid metabolism regulators, and novel genes with unknown functions.

From the genes identified by the microarray analysis, we selected 12 genes up-regulated in TRAF3^−/−^ B lymphomas for further verification by quantitative real time PCR using TaqMan gene expression assay kits. Our data verified the mRNA up-regulation of the 12 genes examined, including *Diras2*, *MCC*, *Tbc1d9*, *Ccbp2*, *Btbd14a*, *Sema7a*, *Twsg1*, *Ppap2b*, *TCF4*, *Tnfrsf19*, *Zcwpw1,* and *Abca3* (Figure 1B). Striking up-regulation of these transcripts was verified in the three splenic B lymphoma samples used for microarray analyses (mouse ID: 6983–2, 7041–10, and 7060–8), and also confirmed in three additional splenic B lymphomas (mouse ID: 7140–7, 7140–3 and 5–5) as well as ascites from two cases (mouse ID: 7060–8 and 7140–3; Figure 1B). Thus, up-regulation of these 12 genes is recurrent in B lymphomas spontaneously developed in different individual B-TRAF3^−/−^ mice.

We next surveyed public gene expression databases of human cancers (http://www.oncomine.org) [42] and searched the literature to investigate whether the genes identified in our microarray analyses exhibit alterations in mRNA expression, DNA copy number variation, or mutations in primary human B lymphomas and other cancers. Interestingly, we found that among the up-regulated genes identified in our microarray analysis, *MCC* is most consistently up-regulated in a variety of primary human B cell malignancies, including PEL, CBL, DLBCL, BL, and MM [38–41]. It has been previously shown that *MCC* functions as a tumor suppressor gene in CRCs and hepatocellular carcinoma [20,27,31–35]. However, the functional roles of MCC in lymphocytes or lymphomas remain unknown. In this context, we selected to further explore the expression and function of MCC in B lymphocytes and B cell malignancies in the present study.

### Striking up-regulation of MCC in TRAF3^-/-^ mouse B lymphomas but not in premalignant TRAF3^-/-^ B lymphocytes

**Figure 2:**
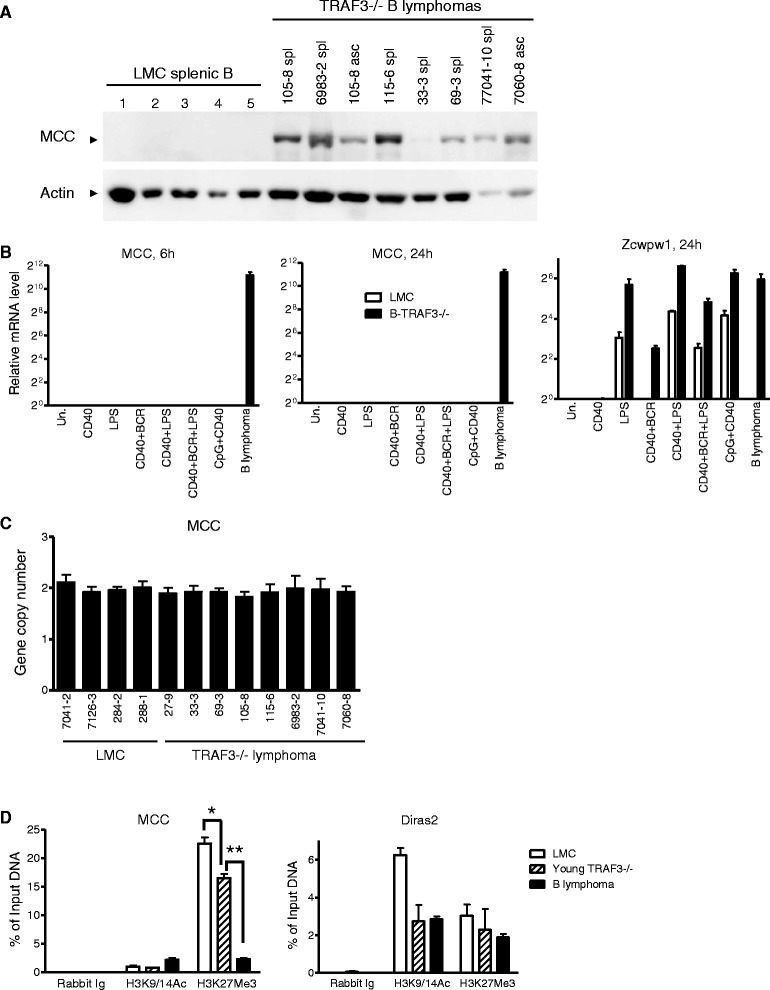
**Striking up-regulation of MCC in TRAF3** ^**-/-**^ **mouse B lymphomas but not in premalignant TRAF3** ^**-/-**^ **B lymphocytes. (A)** Western blot analysis of the MCC protein. Total cellular proteins were prepared from purified LMC splenic B cells or splenic B lymphomas (spl) or ascites (asc) of different individual B-TRAF3^−/−^ mice. **(B)** Regulation of the expression of *MCC* and *Zcwpw1* in response to B cell stimuli. LMC and premalignant (young) TRAF3^-/-^ splenic B cells were cultured *ex vivo* in the absence or presence of stimuli of B cell proliferation, differentiation and activation for 6 or 24 hours. B cell stimuli examined include: 2 μg/ml anti-CD40, 20 μg/ml LPS, 1 μg/ml anti-BCR, and 100 nM CpG2084, alone or in combination. RNA samples of TRAF3^-/-^ B lymphomas (mouse ID: 7060–8) were used as positive control of the *MCC* and *Zcwpw1* transcripts in Taqman assays. **(C)** Normal copy number of the *MCC* gene in TRAF3^-/-^ mouse B lymphomas. Copy number of the mouse *MCC* gene in genomic DNA samples prepared from LMC splenocytes or TRAF3^−/−^ B lymphomas was determined using the TaqMan Copy Number Assay kit. Mouse ID was indicated in the figure. **(D)** Histone modifications of the promoter region of the *MCC* and *Diras2* genes. Chromatin was prepared from LMC and young TRAF3^-/-^ splenic B cells, or TRAF3^-/-^ B lymphomas. Fragmented chromatin was immunoprecipitated using antibodies specific for histone marks (H3K27me3 or H3K9/14ac) or non-specific rabbit Ig. Immunoprecipitated DNA was quantified by qPCR using primer pairs specific for the promoter region of the *MCC* and *Diras2* genes, respectively. Quantity of immunoprecipitated DNA is presented as percentage of input DNA in graphs. All graphs (B-D) depict the results of three independent experiments with duplicate reactions in each experiment (mean ± S.D.). *P < 0.05, and **P < 0.0001 by Student’s *t* test.

We first verified the up-regulation of *MCC* in splenic B lymphomas and ascites spontaneously developed in 8 different individual B-TRAF3^−/−^ mice at the protein level using Western blot analysis (Figure 2A). In contrast, MCC protein expression was not detected in LMC splenic B cells (Figure 2A). We also compared the transcript expression of *MCC* in different mature B cell subsets or B cells of different developmental stages by surveying the public gene expression database of Mouse Immune Genome (http://www.immgen.org). Mature B cell subsets examined include follicular, marginal zone, germinal center, and B1 B cells, and developing B cells examined include common lymphoid progenitor, pre-pro-B, pro-B, pre-B, newly-formed B, and transitional (T1, T2 and T3) B cells. The data in Mouse Immune Genome indicate that the *MCC* transcript is barely detected in any mature B cell subsets or developing B cells of any developmental stages. It has been previously shown that the expression of MCC is gradually up-regulated during differentiation of PC12 cells induced by NGF [37]. We thus investigated the potential up-regulation of *MCC* during the proliferation, differentiation, and activation of B lymphocytes induced by a variety of B cell stimuli. We purified splenic B cells from LMC or tumor-free young B-TRAF3^-/-^ mice (age: 10–12 weeks; premalignant TRAF3^−/−^ splenic B cells), and then stimulated the cells with various B cell stimuli. These include agonistic anti-CD40 Abs, LPS (TLR4 agonist), B cell receptor (BCR) crosslinking Abs, and CpG2084 (TLR9 agonist), alone or in combination. TLR4 and TLR9 were examined as a representative of plasma membrane- and endosome- localized members of the TLR family, respectively [43]. We found that the transcript of *MCC* was neither up-regulated nor detected in LMC or premalignant TRAF3^−/−^ splenic B cells after treatment with any of the stimuli examined (Figure 2B). In contrast, the expression of *Zcwpw1*, another gene identified by our microarray analysis, was robustly up-regulated in LMC or premalignant TRAF3^−/−^ splenic B cells after treatment with LPS alone, LPS in combination with CD40, or CpG in combination with CD40. This indicates that expression of *Zcwpw1* is induced during the proliferation and activation of B lymphocytes. However, unlike *Zcwpw1*, the expression of *MCC* is only up-regulated and detected in TRAF3^−/−^ B lymphomas, suggesting that MCC up-regulation is selectively associated with B cell malignant transformation.

We next cloned the full-length coding sequence of the MCC transcripts from primary splenic B lymphomas of 4 different individual B-TRAF3^−/−^ mice (mouse ID: 6983–2, 7060–8, 105–8, and 115–6) by reverse transcription and PCR. Our sequencing results demonstrated that the *MCC* gene expressed in TRAF3^−/−^ B lymphomas is predicted to encode a protein of 828 amino acids and does not contain any mutations. In an effort to understand how *MCC* is up-regulated in TRAF3^−/−^ B lymphomas, we first determined the copy number of the MCC gene in primary splenic B lymphomas of 8 different individual B-TRAF3^−/−^ mice using Taqman Copy Number Assay Kit. Our results revealed that the copy number of the MCC gene was not changed in TRAF3^−/−^ mouse B lymphomas as compared to that observed in LMC splenocytes (Figure 2C). We next investigated the involvement of epigenetic alterations in MCC up-regulation by performing chromatin immunoprecipitation (ChIP) analyses. We used antibodies specific for a repressive histone mark (trimethylated Lys 27 of histone 3, H3K27Me3) and an activating histone mark (acetylated Lys 9/14 of histone 3, H3K9/14Ac), respectively. We chose to examine these two epigenetic modifications based on previous evidence that alterations of H3K27Me3 and H3K9/14Ac frequently occur in human B lymphomas and MM [44–51]. Our results showed that the activating H3K9/14 acetylation of the promoter region of the *MCC* gene was not obviously changed in TRAF3^−/−^ B lymphoma cells as compared to LMC or premalignant TRAF3^−/−^ splenic B cells (Figure 2D). In contrast, the repressive H3K27 trimethylation of the *MCC* promoter region was almost completely abolished in TRAF3^−/−^ B lymphoma cells, and also modestly decreased in premalignant TRAF3^−/−^ splenic B cells (Figure 2D). Such changes in H3K27 trimethylation was not observed in the promoter region of *Diras2* (Figure 2D), another gene strikingly up-regulated in TRAF3^−/−^ B lymphomas identified in our study. The trimethylation of H3K27 that is almost completely abolished in the *MCC* promoter region in TRAF3^−/−^ B lymphomas is intriguing, considering that the expression levels and activity of EZH2 of the polycomb repressive complex 2 (PRC2) catalyzing H3K27Me3 are often elevated in human B lymphomas and MM [45–47]. This unexpected decrease of the repressive H3K27Me3 of the *MCC* promoter region may be caused by increased activity of the H3K27 demethylase UTX or altered recognition of the *MCC* promoter by PRC2 in TRAF3^−/−^ B lymphoma cells. Altered recognition of the *MCC* promoter by PRC2 might be brought about by changes in DNA methylation of this region, accessory proteins of the PRC2 complex, or expression of EZH2-binding non-coding RNA surrounding the MCC gene locus in TRAF3^−/−^ B lymphomas [47,52–54]. Regardless of the exact mechanisms, our results suggest that alterations in epigenetic modifications (such as H3K27Me3) of the *MCC* promoter region contribute at least partially to the up-regulation of MCC observed in TRAF3^−/−^ B lymphomas.

### Aberrant up-regulation of MCC in human patient-derived MM cell lines with TRAF3 deletions or relevant mutations

**Figure 3:**
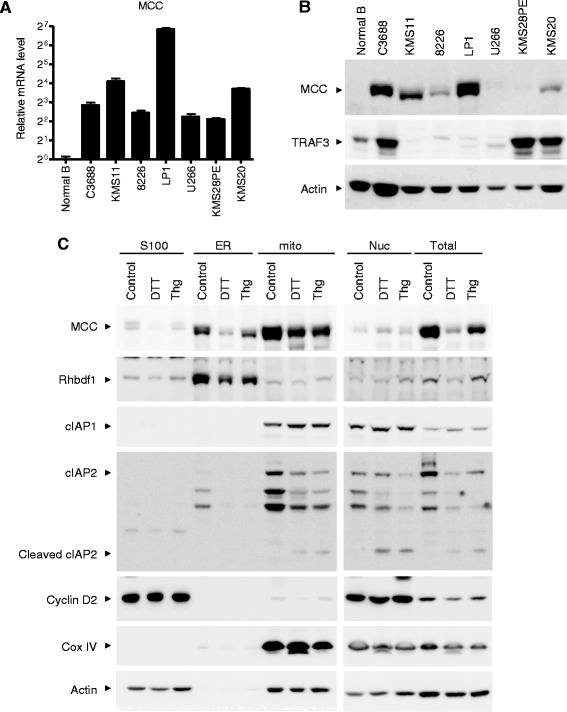
**Aberrant expression and subcellular localization of MCC in human MM cells. (A)** Taqman assay of the *MCC* transcript. Total cellular RNA was prepared from normal human blood B lymphocytes (Normal B), an EBV-transformed B lymphoblastoid cell line C3688, or human MM cell lines with TRAF3 deletions or relevant mutations. The human MM cell lines include KMS11, 8226, LP1, U266, KMS28PE, and KMS20. Real-time PCR was performed using TaqMan primers and probes (FAM-labeled) specific for human *MCC*. Each reaction also included the probe (VIC-labeled) and primers for human β-actin mRNA, which served as an endogenous control. Relative mRNA expression levels of the *MCC* gene were analyzed using the Sequencing Detection Software (Applied Biosystems) and the comparative Ct (ΔΔCt) method. Graphs depict the results of three independent experiments with duplicate reactions in each experiment (mean ± S.D.). **(B)** Western blot analysis of the MCC protein. Total cellular proteins were prepared from normal B lymphocytes, C3688 cells, or human MM cell lines with TRAF3 deletions or relevant mutations. Proteins were immunoblotted for MCC, followed by TRAF3 and actin. Data shown are representative of 3 experiments. **(C)** Subcellular localization of MCC and its regulation during ER stress responses. LP1 cells were cultured in the absence (control) or presence of ER stress inducers DTT or thapsigargin (Thg). After treatment for 24 hours, cytosol (S100), ER, mitochondria (mito) and nuclei (Nuc) were biochemically fractionated from cells, and an aliquot of cells was used for total protein lysates (Total). Proteins in each fraction or total lysates were immunoblotted for MCC, Rhbdf1 (an ER protein), cIAP1, cIAP2, cyclin D2, COX IV (a mitochondrial protein), and actin. Results shown are representative of 3 independent experiments.

To confirm the clinical relevance of our study, we examined *MCC* expression in 6 human patient-derived MM cell lines with TRAF3 deletions or relevant mutations. These include: two cell lines with TRAF3 bi-allelic deletions (KMS11 and 8226), two cell lines with TRAF3 frameshift mutations (LP1 and U266), and two cell lines with cIAP1/2 bi-allelic deletions (KMS28PE and KMS20). Similar to TRAF3 inactivation, bi-allelic deletions of cIAP1/2 also lead to constitutive activation of the non-canonical NF-κB pathway, NF-κB2 (p52/RelB) [9]. We found that MCC is aberrantly up-regulated in all examined human MM cell lines at both the transcript and protein levels (Figure 3A and B). Robust expression of MCC was also detected in an EBV-transformed B lymphoblastoid cell line C3688 [55], which also exhibits constitutive NF-κB2 activation. In contrast, MCC expression was not detected in normal human blood B lymphocytes. It should be noted that MM is the tumor of terminally differentiated B cells, plasma cells. By directly comparing with purified normal plasma cells, microarray analyses by Zhan *et al.* have previously identified aberrant up-regulation of the *MCC* transcript in MM patient samples [39,40]. Similarly, microarray analyses by Basso *et al*. have demonstrated aberrant up-regulation of the *MCC* transcript in patient samples of B lymphomas (PEL, CBL, BL and DLBCL) as compared with normal B cells of different activation stages, including naive pre-germinal center B cells, centroblasts, centrocytes, and memory B cells [38]. Together, the above evidence indicates that *MCC* is aberrantly up-regulated only in malignant B cells and neoplastic plasma cells, but not in their normal counterparts.

We then cloned the full-length coding sequence of the MCC transcripts from LP1 and KMS11 cells by reverse transcription and PCR. Sequencing results of the PCR products of MCC transcripts demonstrated that the coding cDNAs of the MCC gene cloned from human MM cells are predicted to encode a protein of 829 amino acids and contain no mutations. Our results thus demonstrate that MCC is aberrantly expressed in both mouse and human transformed B cells, but not expressed in normal B lymphocytes.

### Subcellular localization of MCC and its regulation in human MM cells during ER stress responses

Subcellular localization of a protein provides important clues about its potential function. It has been previously shown that MCC is localized in the cytosol as well as in the nucleus, and is also associated with the plasma membrane and membrane organelles in epithelial cells [20,31,33,37]. To elucidate where MCC exerts its roles in malignant B cells, we examined the subcellular localization of MCC using a biochemical fractionation method. To investigate whether MCC protein level is regulated in response to apoptosis induction and ER stress, we employed two ER stress inducers, DTT (a chemical that reduces disulfides to thiols and thus affects protein folding or conformation) and thapsigargin (an inhibitor of ER Ca^2+^ transport). We prepared the cytosol, microsomes (rich in ER), mitochondria and nuclei fractions from human MM cells in the absence or presence of treatment with DTT or thapsigargin. Our results revealed that MCC protein was primarily localized in the mitochondria, but also detectable in the ER, cytosol and nucleus in human MM cells (Figure 3C). Interestingly, we noticed that the two ER stress inducers, DTT and thapsigargin, markedly inhibited the protein levels of MCC, but did not change the subcellular localization of MCC in human MM cells (Figure 3C). The ER stress responses induced by DTT and thapsigargin were evident as demonstrated by the cleavage of the cellular inhibitor of apoptosis, cIAP2, and the induction of apoptosis in human MM cells (Figure 3C and data not shown). Yang *et al*. have previously shown that cIAP2 is cleaved by the mitochondrial serine protease Omi/HtrA2 during apoptosis [56]. Given the predominant mitochondrial localization of MCC, it is likely that the ER stress-induced decrease of MCC protein levels may be mediated through selective mitochondrial autophagy or proteasome-dependent degradation [57–59]. Together, our data demonstrated that MCC exhibits a primary mitochondrial localization in human MM cells, and that MCC protein levels are down-regulated by ER stress and apoptosis induction in malignant B cells. These results suggest that MCC may play a role in regulating malignant B cell survival and proliferation.

### Lentiviral shRNA vector-mediated knockdown of MCC induced apoptosis and inhibited proliferation in human MM cells

Previous studies have shown that MCC blocks cell cycle progression and inhibits cell proliferation in fibroblasts and CRCs [20,31,32,35]. To explore the functional roles of MCC in regulating malignant B cell survival and proliferation, we employed genetic means to manipulate the expression levels of MCC in human MM cells, including lentiviral vector-mediated overexpression and knockdown of MCC, respectively. In contrast to the cell cycle blocking and proliferation inhibitory effects of MCC overexpression reported in fibroblasts and CRCs [20,31,32,35], we found that overexpression of MCC in human MM cell line 8226 cells, which express endogenous MCC at relatively modest levels, did not affect cell cycle progression, cell proliferation, or cell survival at all (data not shown). It has been previously shown that the expression level of ectopically transfected MCC was strongly reduced in CRCs after several passages, suggesting selection against MCC expression in CRC cultures [35]. However, we observed that overexpression of MCC was maintained in human MM 8226 cells after >10 passages. Together, the results of our overexpression studies argue against a negative role for MCC in the survival or proliferation of malignant B cells.

**Figure 4:**
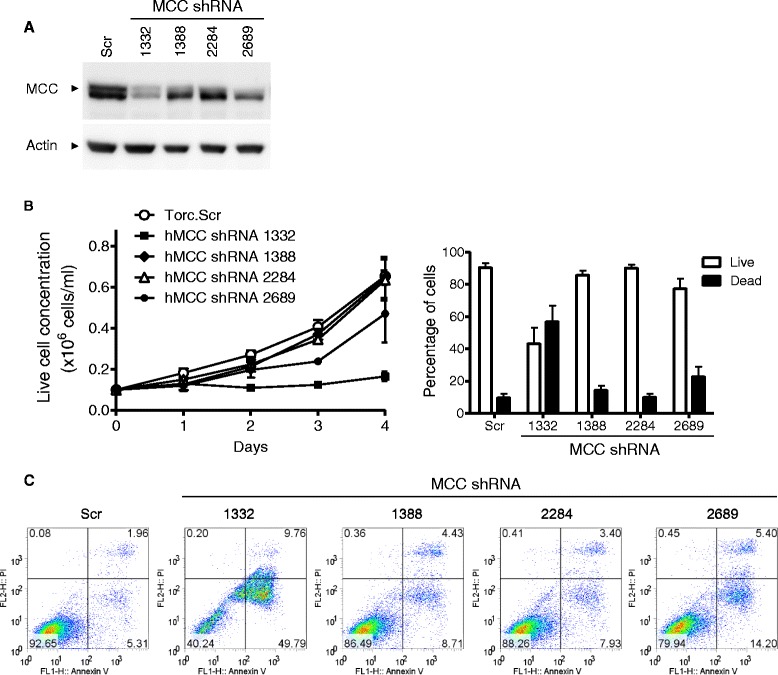
**Lentiviral shRNA vector-mediated knockdown of MCC induced apoptosis in human MM cells.** The human MM cell line LP1 cells were transduced with lentiviruses expressing MCC shRNAs (including 1332, 1388, 2284 or 2689) or a scrambled shRNA (Scr). **(A)** Knockdown of the MCC protein determined by immunoblot analysis. Total cellular proteins were prepared from LP1 cells successfully transduced with a scrambled shRNA (Scr), or MCC shRNA 1332, 1388, 2284 or 2689 on day 5 post-transduction. Proteins were immunoblotted for MCC, followed by actin. Immunoblot of actin was used as a loading control. Results shown are representative of 3 independent experiments, and similar results were obtained in the human MM cell line KMS11 cells. **(B)** Growth curves of live cells and percentage of live versus dead cells determined by Trypan blue-stained cell counting. On day 4 post-transduction, successfully transduced LP1 cells were cultured in a 6-well plate for growth curve determination. Live and dead cells were counted daily for 4 days using Trypan blue staining and a hemocytometer. The graphs depict the results of 3 independent experiments (mean ± S.D.). **(C)** Representative FACS profiles of transduced cells analyzed by annexin V and PI staining. On day 6 post-transduction, transduced LP1 cells were stained with annexin V and PI, and then analyzed by a flow cytometer. In the FACS profiles, apoptotic cells were identified as annexin V+ PI-, dead cells were annexin V+ PI+, and live cells were annexin V- PI-. Data shown are representative of 3 independent experiments, and similar results were obtained in the human MM cell line KMS11 cells.

**Figure 5:**
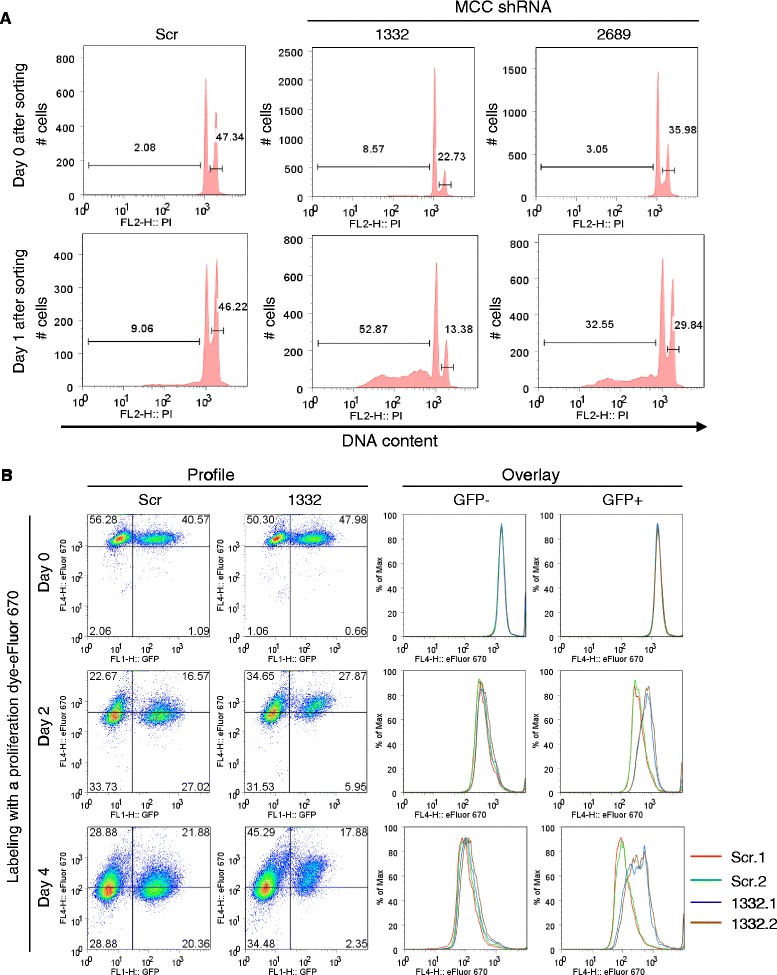
**Knockdown of MCC induced apoptosis and inhibited proliferation in human MM cells.** **(A)** Cell cycle distribution analyzed by PI staining and flow cytometry. The human MM cell line LP1 cells were transduced with lentiviruses expressing MCC shRNAs (1332 or 2689) or a scrambled shRNA (Scr). Successfully transduced (GFP+) cells were sorted by a FACS sorter on day 4 post-transduction. Top panel shows the FACS histograms of cells right after sorting (Day 0), and bottom panel shows the FACS histograms of sorted cells after cultured for 24 hours (Day 1). Gated populations in the FACS histograms indicate apoptotic cells (DNA content < 2n) or proliferating cells (2n < DNA content ≤ 4n). Results shown are representative of 3 independent experiments, and similar results were also obtained in the human MM cell line KMS11 cells. **(B)** Cell proliferation analyzed by dilution of the proliferation dye (eFluor 670)-labeling and flow cytometry. On day 4 post transduction, LP1 cells transduced with MCC shRNA 1332 (1332) or a scrambled shRNA (Scr) were labeled with a proliferation dye eFluor 670, which binds to any cellular protein containing primary amines. As cells divide, the dye is distributed equally between daughter cells that can be measured as successive halving or dilution of the fluorescence intensity of the dye. Day 0 FACS profiles and overlay histograms show the eFluor 670 signals of freshly labeled cells, while those of Day 2 and Day 4 show diluted eFluor 670 signals of cells after cultured for 2 and 4 days, respectively. Inhibited proliferation of MCC shRNA 1332-transduced cells was demonstrated by the hampered dilution of the proliferation dye as compared to those observed in both the untransduced (GFP-) cells and the scrambled shRNA (Scr)-transduced cells. Results shown are representative of 3 independent experiments with duplicate samples in each experiment.

We next screened four lentiviral shRNA vectors specific for human *MCC* using the human MM cell line LP1 cells. Cells transduced with a scrambled shRNA vector were used as control in these experiments. Our results of Western blot analysis showed that the MCC shRNA 1332 knocked down the protein levels of MCC by ~85% reduction, while the MCC shRNA 2689 decreased MCC levels by ~70% reduction in LP1 cells (Figure 4A). We subsequently used these MCC shRNA lentiviruses to transduce human MM cell lines LP1 and KMS11 cells. We found that both MCC shRNAs 1332 and 2689 inhibited growth and induced apoptosis in human MM cells, as demonstrated by growth curve determination, trypan blue or PI staining of dead cells, and annexin V staining of apoptotic cells (Figure 4B and C). Interestingly, MCC shRNA 1332 that resulted in a greater decrease in MCC protein level was also most effective at inducing apoptosis in human MM cells. In contrast, MCC shRNAs 1388 and 2284, which did not markedly knock down MCC protein level, did not drastically induce apoptosis in human MM cells (Figure 4). We further determined whether knockdown of MCC inhibits the proliferation of human MM cells by performing cell cycle analysis and proliferation dye labeling experiments. Results of cell cycle analysis demonstrated that transduction of MCC shRNA 1332 or 2689 resulted in a dramatic decrease in the proliferating cell population (S/G2/M phase: 2n < DNA content ≤ 4n) and an increase in the apoptotic cell population (DNA content < 2n) in human MM cells (Figure 5A). As observed for apoptosis induction, MCC shRNA 1332 was also more potent than shRNA 2689 in decreasing the proliferating cell population of human MM cells (Figure 5A). Our results of the proliferation dye labeling experiments further confirmed that knockdown of MCC by shRNA 1332 remarkably inhibited the proliferation of human MM cells. This was demonstrated both by the reduction of GFP+ shRNA 1332-transduced cells and by the slower dilution of the proliferation dye in shRNA 1332-transduced cells as compared to scrambled shRNA-transduced cells or untransduced cells after culture for 4 days (Figure 5B). Taken together, our results demonstrate that MCC plays a positive role and is required for the survival and proliferation in human MM cells, indicating that MCC acts as an oncogene in B lymphocytes.

### MCC signaling pathways in human MM cells

**Figure 6:**
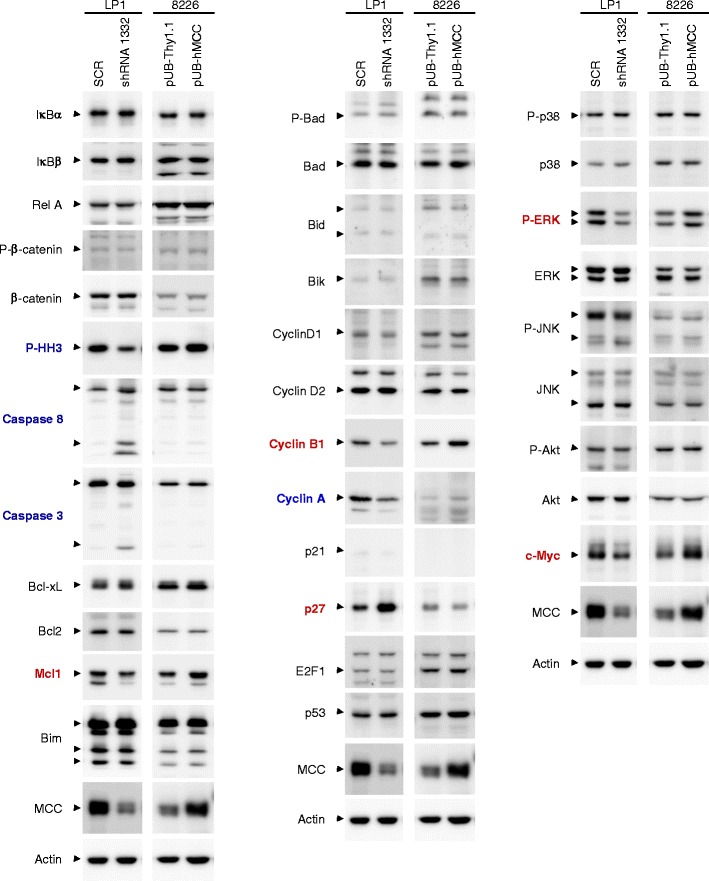
**MCC signaling pathways in human MM cells.** The human MM cell line LP1 cells, which express high levels of endogenous MCC, were transduced with lentiviruses expressing MCC shRNA 1332 or a scrambled shRNA (SCR). Another human MM cell line 8226 cells, which express modest levels of endogenous MCC, were transduced with lentiviruses expressing hMCC (pUB-hMCC) or Thy1.1 only (pUB-Thy1.1). Total cellular lysates were prepared and immunoblotted for known targets of MCC and regulators of apoptosis or cell cycle. Known target proteins of MCC examined include phosphorylated β-catenin (P-β-catenin), β-catenin, IκBα, IκBβ, RelA, and phosphorylated histone H3 (P-HH3). Regulators of apoptosis examined include caspase 8, caspase 3, Bcl-xL, Bcl2, Mcl1, Bim, P-Bad, Bad, Bid, and Bik. Regulators of cell cycle examined include cyclin D1, cyclin D2, cyclin B1, cyclin A, p21, p27, E2F1, p53, phosphorylated-p38 (P-p38), p38, P-ERK, ERK, P-JNK, JNK, P-Akt, Akt, and c-Myc. The MCC blot was used as a control for MCC shRNA 1332 or pUB-hMCC transduction, and the actin blot was used as a loading control. Proteins that are changed by knockdown of MCC are highlighted in blue, and those that are changed by both knockdown and overexpression of MCC are highlighted in red. Results shown are representative of 3 independent experiments.

To understand the mechanisms of MCC shRNA-mediated induction of apoptosis and inhibition of proliferation in human MM cells, we investigated the involvement of known MCC targets. In light of the previous evidence that MCC specifically targets and negatively regulates the oncogenic NF-κB and β-catenin pathways in CRCs and hepatocellular carcinoma [20,27,32,36], we determined the effects of MCC knockdown on the levels of these two pathways in human MM cells, including IκBα, IκBβ, RelA, phospho-β-catenin and β-catenin [20,27,32,36]. Our results showed that knockdown or overexpression of MCC did not change any of the proteins of the NF-κB and β-catenin pathways in human MM cells (Figure 6). We further examined another known MCC target involved in DNA damage response in CRCs, phospho-histone H3 (P-HH3) [35], which is a marker of mitosis. Contrary to what was observed in CRCs [35], we found that knockdown of MCC markedly decreased the level of P-HH3 in human MM cells, confirming the proliferation-promoting function of MCC in malignant B cells.

We then measured the levels or activation of a number of regulators of cell apoptosis and proliferation, including caspases, the Bcl-2 family proteins, and cyclins. Our results demonstrated that knockdown of MCC led to activation of both caspases 8 and 3, as evidenced by their cleavage into active caspase fragments (Figure 6). Interestingly, knockdown of MCC decreased the level of Mcl1 (an anti-apoptotic protein of the Bcl2 family), while overexpression of MCC increased the level of Mcl1 in human MM cells (Figure 6). However, MCC knockdown or overexpression did not affect the protein levels of other members of the Bcl2 family, including Bcl-xL, Bcl2, Bim, Bad, Bid and Bik (Figure 6). Among the cell cycle regulators examined, knockdown of MCC caused specific down-regulation of the protein levels of cyclin B1 and cyclin A, while overexpression of MCC increased the level of cyclin B1 in human MM cells (Figure 6). Knockdown of MCC also led to specific up-regulation of the protein level of the cell cycle inhibitor p27, which was modestly down-regulated by overexpression of MCC in human MM cells (Figure 6). In contrast, MCC knockdown did not change other cell cycle regulators examined, including cyclin D1, cyclin D2, p21, E2F1, and p53 (Figure 6). Our findings suggest that MCC inhibits apoptosis and induces proliferation by inhibiting cleavage of caspases 8 and 3, up-regulating Mcl1 and cyclin B1, and down-regulating p27 in human MM cells.

To further understand the mechanisms of MCC-mediated regulation of cell survival and proliferation, we sought to investigate key signaling pathways that are known to play important roles in regulating B cell survival and proliferation, including the activation of p38, ERK, JNK, Akt and c-Myc [60]. Our results showed that knockdown of MCC led to specific decreases in the phosphorylation level of ERK1/2 and the protein level of c-Myc (a downstream target of ERK), while overexpression of MCC exhibited the opposite effect in human MM cells (Figure 6). In contrast, knockdown or overexpression of MCC did not change the activation of p38, JNK and Akt in human MM cells (Figure 6). These results indicate that ERK-c-Myc is a key signaling pathway underlying the oncogenic roles of MCC in malignant B cells.

### MCC interacting proteins in human MM cells

To gain further insights into the molecular mechanisms underlying the oncogenic roles of MCC in B cells, we set out to identify MCC interacting proteins in human MM cells. To facilitate co-immunoprecipitation studies, we constructed two lentiviral expression vectors of tagged human MCC, including pUB-FLAG-hMCC and pUB-hMCC-SBP-6×His, which express an N-terminal FLAG tagged or a C-terminal SBP-6×His tagged MCC, respectively. We used these vectors to transduce human patient-derived MM cell line 8226 cells, which express endogenous MCC at relatively modest levels. Our flow cytometric data demonstrated that the lentiviral transduction efficiency by pUB-FLAG-hMCC and pUB-hMCC-SBP-6×His was > 85% in 8226 cells (data not shown). Transduced FLAG-hMCC and hMCC-SBP-6×His were immunoprecipitated from whole cell lysates using anti-FLAG-agarose or streptavidin-sepharose beads, respectively. We first examined a number of previously known MCC interacting proteins identified in CRCs or 293T cells, including β-catenin, Mst3, VCP, PP2A, DFFA, VHL, VDAC, scribble, and myosin IIb [20,32–34,61]. However, none of these proteins were detected in co-immunoprecipitates with either FLAG-MCC or MCC-SBP-6×His in human MM cells. Thus, our findings suggest that MCC inhibits apoptosis and promotes proliferation likely through interaction with novel, previously unknown partners in malignant B cells.

**Figure 7:**
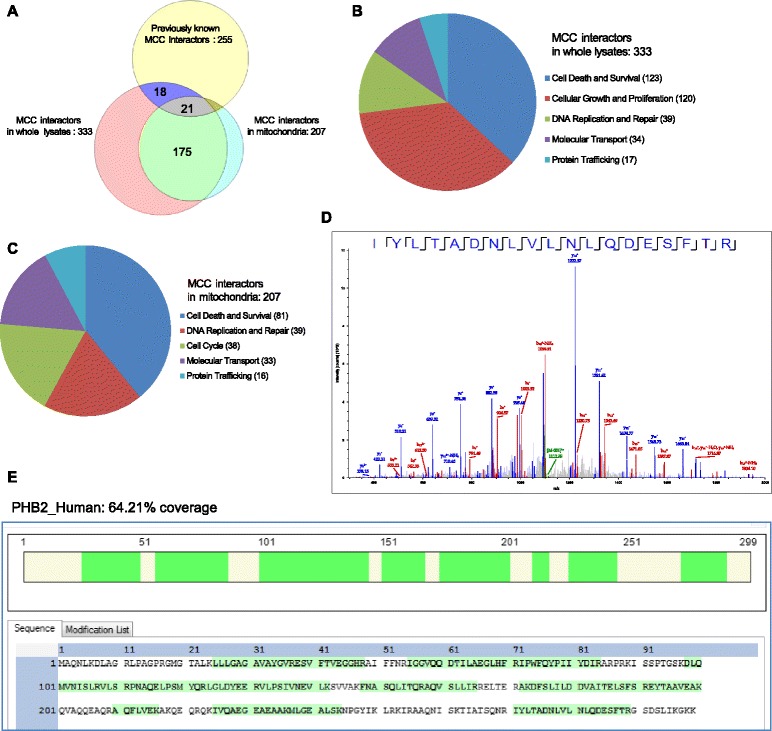
**The MCC-interactome in human MM cells identified by affinity purification followed by LC-MS/MS.** The human MM cell line 8226 cells were transduced with lentiviruses expressing hMCC-SBP-6xHis or FLAG-hMCC. Immunoprecipitates of hMCC-SBP-6xHis by streptavidin-sepharose beads from whole cell lysates and purified mitochondria of 8226 cells were analyzed by high resolution LC-MS/MS, respectively. Immunoprecipitates of FLAG-hMCC by streptavidin-sepharose beads were used as negative control in these experiments. **(A)** Venn diagram of MCC-interactors identified in the present study and previous studies. The diagram depicts the number of previously known MCC-interactors or MCC-interactors identified in whole cell lysates or mitochondria of human MM cells in our study, as well as the overlap between each category. **(B and C)** Functional clustering and annotation of MCC-interactors identified in whole cell lysates **(B)** or mitochondria **(C)** of human MM cells. The number of proteins of each functional cluster is shown. **(D)** Representative mass spectral profile of a peptide of PHB2 identified by LC-MS/MS. The deduced peptide sequence is shown. **(E)** Schematic diagram of peptide sequences of PHB2 identified by LC-MS/MS. The peptide sequences detected by LC-MS/MS are highlighted in green color.

To delineate the novel MCC-interactome in human MM cells, we turned to employ an unbiased strategy, affinity purification followed by mass spectrometry-based sequencing. Considering the primary mitochondrial localization of MCC in human MM cells, immunoprecipitates of hMCC-SBP-6×His by streptavidin-sepharose beads from both whole cell lysates and purified mitochondria of 8226 cells were analyzed by high resolution LC-MS/MS, respectively. In these experiments, immunoprecipitates of FLAG-hMCC by streptavidin-sepharose beads were used as negative control. Using this strategy, we identified 365 proteins in the MCC-interactome of human MM cells: 333 MCC interacting proteins in whole cell lysates, and 207 MCC interactors in mitochondria (Figure 7A, and Additional file 2: Table S2). Among these, 175 MCC interactors were identified in both whole cell lysates and purified mitochondria of human MM cells. However, only 39 (out of 365, 10.7%) proteins of our study were also previously identified as MCC interacting proteins in CRCs or 293 T cell (Figure 7A, and Additional file 2: Table S2) [20,32–34,36,61]. Thus, most of the proteins identified in our study are novel MCC-interacting partners.

We next performed disease association analyses using Ingenuity (http://www.ingenuity.com), and cancer is identified as the top disease associated with the MCC-interactome identified in our study. Results of Ingenuity analyses showed that 195 of the 333 (58.5%) MCC interactors in whole cell lysates and 91 of the 207 (43.96%) MCC interactors in mitochondria are associated with cancer. Functional annotation and clustering analyses by Ingenuity revealed that mitochondrial MCC interactors are mainly regulators of cell death and survival (81 of 207, 39.1%) (Figure 7B). Similarly, MCC interactors in whole cell lysates are mainly regulators of cell death and survival (123 of 333, 36.9%) or cellular growth and proliferation (120 of 333, 36%) (Figure 7C). Other MCC interactors include regulators of DNA replication and repair, molecular transport, and protein trafficking (Figure 7B and C). Together, the MCC-interactome identified in our study is consistent with the prominent roles of MCC in promoting survival and proliferation in human MM cells (Figures 4 and 5).

There are two isoforms of *MCC* encoding proteins that differ at their extreme N-terminus due to alternative promoter usage, isoform 1 (828 amino acids in mouse and 829 amino acids in human) and isoform 2 (1019 amino acids). The *MCC* that we cloned from LP1 cells and used to generate FLAG-hMCC and hMCC-SBP-6×His is isoform 1 (829 aa). Interestingly, our LC-MS/MS data identified 6 unique peptides of the isoform 2 of MCC (1019 aa) in the immunoprecipitates of hMCC-SBP-6×His in both whole cell lysates and purified mitochondria (Additional file 3: Figure S1 and Additional file 2: Table S2). These results demonstrate that the two isoforms of MCC form hetero-dimers or hetero-oligomers in human MM cells.

**Figure 8:**
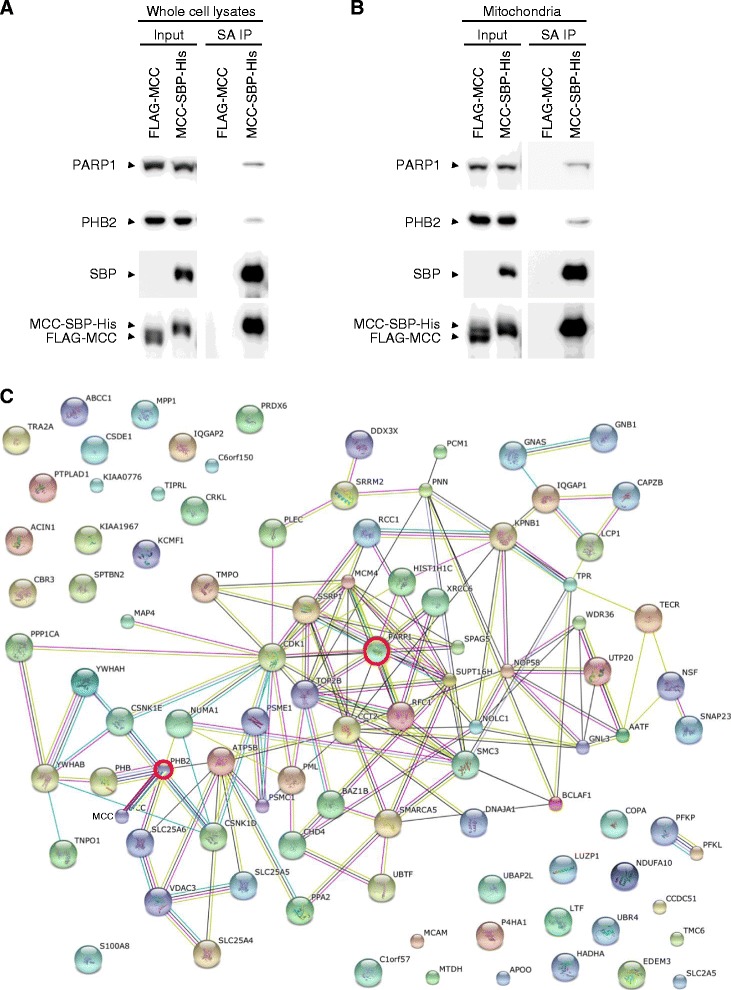
**PARP1 and PHB2 are two hubs of the MCC interaction network in human MM cells.** The human MM cell line 8226 cells were transduced with pUB-hMCC-SBP-6xHis or pUB-FLAG-hMCC. Immunoprecipitation was performed as described in Figure 7. **(A and B)** Immunoblot analyses of proteins pulled down by streptavidin-sepharose beads (SA IP) from whole cell lysates **(A)** or purified mitochondria **(B)**. Proteins were immunoblotted for PARP1, PHB2, SBP, and followed by MCC. Blots of an aliquot of whole cell or mitochondrial lysates before immunoprecipitation were used as the input control. **(C)** The MCC interaction network in human MM cells**.** We performed an interaction network analysis using the top 100 MCC-interactors and the STRING analysis tool (http://www.string-db.org). The top 100 MCC-interactors identified in our study and used for the interaction network analysis include 60 proteins identified in both whole cell lysates and mitochondria, 10 proteins identified in mitochondria only, and 30 proteins identified in whole cell lysates only. Sixty-one proteins of the top 100 MCC-interactors of human MM cells form an interaction network centered at PARP1 and PHB2.

From the top 10 MCC-interactors identified in both whole cell lysates and purified mitochondria of human MM cells, we selected to further verify the interaction of MCC with two important regulators of cell survival and proliferation, PARP1 and prohibitin-2 (PHB2, Figure 7D and E) [62–65], by co-immunoprecipitation and Western blot analyses. Our results confirmed the interactions of MCC with PARP1 and PHB2 in both whole cell lysates and purified mitochondria of human MM cells (Figure 8A and B). We further performed an interaction network analysis using the top 100 MCC-interactors of human MM cells (60 proteins identified in both whole cell lysates and mitochondria, 10 proteins identified in mitochondria only, and 30 proteins identified in whole cell lysates only) and the STRING analysis tool (http://www.string-db.org) [66], which builds protein interaction networks based on known and predicted protein-protein interactions. As shown in Figure 8C, 61 proteins of the top 100 MCC-interactors form an interaction network centered upon PARP1 and PHB2. Interestingly, PARP1 and PHB2 have also been previously shown to directly or indirectly interact with and/or regulate MCC targets identified by knockdown and overexpression of MCC studies in human MM cells (Figure 6), including ERK, c-Myc, p27, cyclin B1, Mcl-1, caspase 8, and caspase 3 [62–65]. Taken together, our findings indicate that MCC promotes cellular survival and proliferation by associating with and modulating the interaction network centered at PARP1 and PHB2 in malignant B cells.

## Discussion

The late onset and long latency of lymphoma development observed in B-TRAF3^-/-^ mice suggest that secondary oncogenic hits are required to promote B cell transformation. In the present study, we identified *MCC* as a gene strikingly up-regulated in TRAF3^−/−^ mouse B lymphomas and human MM cell lines. Aberrant expression of *MCC* has also been documented in primary human B cell malignancies, including PEL, CBL, DLBCL, BL, and MM [38–41]. In contrast, *MCC* expression was not detected in normal or premalignant TRAF3^−/−^ B cells even after treatment with B cell stimuli, suggesting that aberrant expression of *MCC* is specifically associated with malignant transformation of B cells. In elucidating the function of MCC in malignant B cells, we found that lentiviral shRNA-mediated knockdown of MCC induced apoptosis and inhibited proliferation in human MM cells. Experiments of knockdown and overexpression of MCC allowed us to identify downstream targets of MCC in human MM cells, including ERK, c-Myc, p27, cyclin B1, Mcl-1, caspase 8, and caspase 3. Furthermore, we delineated the profile of proteins assembled in the MCC signaling complex in whole cells or mitochondria by employing affinity purification followed by mass spectrometry-based sequencing. Our results indicate that MCC associates with the interaction network centered upon PARP1 and PHB2 to promote cellular survival and proliferation in malignant B cells. Collectively, our findings indicate that MCC functions as an oncogene in B cells.

Paradoxically, in contrary to the up-regulation of *MCC* in B cell neoplasms, the *MCC* gene is frequently deleted, mutated, or silenced in other human cancers, including colorectal cancer [14–17,19,20,22], lung cancer [17,23], gastric carcinoma [24], esophageal cancer [25], and hepatocellular carcinoma [26,27]. Functional evidence and signaling pathway studies indicate that *MCC* acts as a tumor suppressor gene by inhibiting the oncogenic NF-κB and β-catenin pathways in CRCs and hepatocellular carcinoma [20,27,31–36]. We also previously observed regulation of β-catenin by Sox5, another gene identified in our microarray analysis, in human MM cells [67]. However, NF-κB and β-catenin levels were not altered by knockdown or overexpression of MCC in human MM cells. Thus, MCC plays distinct roles via different signaling mechanisms in B cell malignancies versus other human cancers.

It has been shown that in CRCs, MCC mainly localizes in the cytoplasm, and is induced to shuttle into the nucleus in response to DNA damage [35]. Interestingly, we found that MCC is primarily localized at mitochondria, but also detectable in the ER, cytosol and nucleus in human MM cells. MCC does not contain any mitochondrial targeting motifs or transmembrane domains. Through delineation of the mitochondrial MCC-interactome, we identified a number of mitochondrial proteins that are associated with MCC in human MM cells, including PHB2, prohibitin (PHB), ECHA, VDAC3, ADT1, ADT2, and ADT3. All of these mitochondrial proteins are known as critical regulators of cell survival and apoptosis (http://www.ingenuity.com). Therefore, MCC is primarily localized at mitochondria to promote cellular survival by interacting with multiple mitochondrial proteins in malignant B cells.

Most proteins (326 out of 365, 89.3%) of the MCC-interactome identified in our study are novel, previously unknown MCC-interacting partners. Among these, PARP1 is the top novel MCC-interactor identified in both mitochondrial and whole cell lysates of human MM cells. PARP1, the most abundant member of the polyADP-ribose polymerases (PARP) family, catalyzes post-translational modification of proteins by polyADP-ribosylation. This modification affects protein-protein and protein-DNA interactions. In addition to its pivotal role in DNA repair, PARP1 also critically regulates transcription, cell survival, and proliferation [62,63]. PARP1 can act in both a pro- and anti-tumor manner depending on the context [62]. In this study, we identified PARP1 as a signaling hub of the MCC-interactome in human MM cells (Figure 8C). PARP1 is known to interact with numerous MCC interactors identified in our study, including CDK1, MCM4, XRCC6, RFC1, CCT2, SSRP1, TOP2B, HIST1H1C, and SUPT16H (http://www.string-db.org). Interestingly, PARP1 has been shown to directly or indirectly regulate the activation or expression of multiple MCC targets identified by our knockdown and overexpression experiments, including caspase 8, ERK, c-Myc, p27, cyclin B1, and Mcl1 (Figure 6). Indeed, PARP1 can induce the PARylation of caspase-8, thereby inactivating caspase-8 and inhibiting caspase-mediated apoptotic signaling [62]. PARP1 directly interacts with phosphorylated ERK2 to mediate cell proliferation, whereas PARP1 inhibition causes loss of ERK2 stimulation [62,63]. PARP1 also down-regulates the expression of MKP-1, which dephosphorylates ERK [62]. Through modifications of chromatin or interactions with gene specific promoters/transcription factors, PARP1 regulates in total 3.5% of the transcriptome, including increasing the expression of c-Myc [62,63] and repressing the expression of p27 [68]. Furthermore, PARP1 can indirectly regulate the degradation and anti-apoptotic function of Mcl1 through interaction with CDK1/cyclin B1 [69,70]. Therefore, PARP1 is a novel MCC-interactor that plays a central role in mediating the oncogenic effects of MCC in malignant B cells.

Thirty-nine proteins of the MCC-interactome identified in our study are previously known as MCC-interacting proteins in CRCs or 293T cells. Among these, PHB2 is the top known MCC-interactor identified in both mitochondrial and whole cell lysates of human MM cells. PHB2, a ubiquitously expressed pleiotropic protein, is mainly localized in mitochondria by forming heteromeric complex with PHB, but also present in the cytosol, nucleus and plasma membrane [64,65,71]. PHB2 plays crucial roles in regulating mitochondrial function, cell survival, proliferation, stress response, and development [64,65,71]. PHB2 is expressed at higher levels in proliferating cells, including neoplastic tissues [64,65,71]. Silencing or abrogation of PHB2 induces apoptosis and reduces proliferation in a variety of cancer cells [64,65,71]. Here we identified PHB2 as another hub of the MCC interaction network in human MM cells (Figure 8C). Previous studies have demonstrated the association between PHB2 and multiple MCC-interactors identified in our study, including PHB, CSNK1D (CK1δ), CSNK1E (CK1ε), ATP5B, NUMA1, and SLC25A6 (http://www.string-db.org) [65,72]. PHB2 has also been shown to directly or indirectly regulate the activity or expression of multiple MCC targets identified in our study, including caspase 3, ERK, c-Myc, p27, and cyclin B1 (Figure 6). For example, over-expression of PHB2 inhibits caspase 3 activation and cell apoptosis, whereas down-regulation of PHB2 is associated with increased caspase 3 expression and cell apoptosis [64,65,71]. PHB and PHB2 interact with c-Raf to induce the phosphorylation of ERK1/2 via MEK1 [65]. Through activation of ERK1/2, PHB2 may also indirectly regulate the expression levels of downstream targets of the ERK pathway, including c-Myc, p27 and cyclin B1 [65,71,73,74]. The c-Myc up-regulation mediated by MCC-PHB2 or MCC-PARP1 via ERK signaling pathway in MM cells is functionally analogous to that induced by canonical Wnt-β-catenin signal activation observed in epithelial cells [75,76]. Thus, similar to PARP1, PHB2 and PHB are likely to play essential roles in mediating the oncogenic effects of MCC in malignant B cells.

Interestingly, preclinical evidence indicates that both PARP1 and PHB2/PHB are excellent therapeutic targets in cancer [62,65]. In preclinical studies, PARP inhibitors (such as olaparib) exhibit potent tumoricidal activities on breast cancer, ovarian cancer, pancreatic cancer, prostate cancer, Ewing’s sarcoma, small cell lung carcinoma, and neuroblastoma, among others. The therapeutic effects of olaparib on BRCA-mutated breast cancer have been confirmed in early phase clinical trials with only mild adverse side effects [62]. Notably, PHB ligands (such as flavaglines and capsaicin) display robust cytotoxicity on cancer cells, but have cytoprotective activities on normal cells (e.g. neurons and cardiomyocytes), particularly against oxidative stress [65]. PHB and PHB2 were recently identified as the direct targets of flavaglines (e.g., rocaglamide, rocaglaol and silvestrol), natural products isolated from medicinal plants that show significant anticancer effects but no sign of toxicity in mice [65]. Capsaicin, a component of hot chili peppers, binds to PHB2, and this binding induces apoptosis in human myeloid leukemia cells [65]. Both flavaglines and capsaicin regulate subcellular localization of PHB2 [77–79]. Flavaglines specifically inhibit PHB1/2-c-Raf interaction and prevent PHB1/2-c-Raf membrane localization [79], while capsaicin induces the translocation of PHB2 from mitochondria to the nucleus [77,78]. Such specific targeting mechanisms of PHB ligands suggest that they may exert robust synergistic effects with conventional chemotherapies that target different signaling pathways, including DNA damage response, proteasome, the Bcl-2 family, and NF-κB activation. In this regard, flavaglines have been shown to enhance the efficacy of doxorubicin in mouse lymphoma models [65]. Therefore, identification of PARP1 and PHB2/PHB as hubs of the signaling pathways of MCC in human MM cells implicates potential use of PARP inhibitors and PHB ligands in the treatment of B cell malignancies involving aberrant expression of MCC.

## Conclusions

In the present study, we have identified MCC as a novel oncogene in B lymphocytes and provided insights into its signaling mechanisms in human MM cells. In the unique cellular context of malignant B cells, MCC forms an interaction network centered at PARP1 and PHB2 to promote cellular survival and proliferation by up-regulating ERK activation, c-Myc, Mcl1, and cyclin B1, and by down-regulating p27 and suppressing cleavage of caspases 8 and 3. The lack of expression of MCC in normal or premalignant B cells but ubiquitous up-regulation of MCC in primary human B cell malignances suggests that MCC may be a useful diagnostic marker for B cell neoplasms. Our finding that knockdown of MCC induced apoptosis and inhibited proliferation in human MM cells suggests that MCC may also serve as a therapeutic target in B cell malignancies. Furthermore, the central role of PARP1 and PHB2 in the MCC interaction network of human MM cells implies that PARP1 inhibitors and PHB ligands may have therapeutic application in B cell neoplasms, including NHL and MM.

## Methods

### Mice, cell lines, and reagents

Mice and disease monitoring were as previously described [12,13]. Human patient-derived MM cell lines were generously provided by Dr. Leif Bergsagel (Mayo Clinic, Scottsdale, AZ), including 8226, KMS11, LP1, U266, KMS28PE, and KMS20. The EBV-transformed human B lymphoblastoid cell line C3688 was provided by Dr. Lori Covey (Rutgers University, Piscataway, NJ). All human B cell lines were cultured as described [80]. Most antibodies and reagents used in this study were as previously described [13,67,80]. Mouse monoclonal Abs to MCC were purchased from Santa Cruz Biotechnology (Santa Cruz, CA). DTT, thapsigargin, and lentiviral shRNA constructs for human MCC were purchased from Sigma-Aldrich Corp. (St. Louis, MO). The plasmid pENTR1A-NTAP-A containing the SBP tag sequence was purchased from Addgene (Cambridge, MA). PARP1 Abs were from eBioscience (San Diego, CA), and PHB2 Abs were purchased from Bethyl Laboratories Inc (Montgomery, TX). Additional polyclonal rabbit Abs were from Cell Signaling Technology (Beverly, MA).

### Transcriptome microarray analysis

Total RNA was extracted from splenocytes of LMC (mouse ID: 6983–6, 7041–9, and 7060–5) and tumor-bearing B-TRAF3^−/−^ mice (mouse ID: 6983–2, 7041–10, and 7060–8) using TRIzol reagent (Invitrogen, Carlsbad, CA) following the manufacturer’s instructions. RNA quality was assessed on an RNA Nano Chip using an Agilent 2100 Bioanalyzer (Agilent Technologies, Palo Alto, CA). The mRNA was amplified with a TotalPrep RNA amplification kit with a T7-oligo(dT) primer according to the manufacturer’s instructions (Ambion), and microarray analysis was carried out with the Illumina Sentrix MouseRef-8 24 K Array at the Burnham Institute (La Jolla, CA). Results were extracted with Illumina GenomeStudio v2011.1, background corrected and variance stabilized in R/Bioconductor using the lumi package [81,82] and modeled in the limma package [83]. Microarray data are available from NIH GEO Accession GSE48818.

### Taqman assays of the transcript expression of identified genes

Complementary DNA (cDNA) was prepared from RNA using High Capacity cDNA Reverse Transcription Kit (Applied Biosystems, Carlsbad, CA). Quantitative real-time PCR of specific genes was performed using corresponding TaqMan Gene Assay kit (Applied Biosystems) as previously described [84]. Briefly, real-time PCR was performed using TaqMan primers and probes (FAM-labeled) specific for mouse *MCC, Diras2*, *Tbc1d9*, *Ccbp2*, *Btbd14a*, *Sema7a*, *Twsg1*, *Ppap2b*, *TCF4*, *Tnfrsf19*, *Zcwpw1*, *Abca3*, or human *MCC*. Each reaction also included the probe (VIC-labeled) and primers for mouse or human β-actin mRNA, which served as an endogenous control. Relative mRNA expression levels of each gene were analyzed using the Sequencing Detection Software (Applied Biosystems) and the comparative Ct method (ΔΔCt) as previously described [84].

### Splenic B cell purification and stimulation

Splenic B cells were purified using anti-mouse CD43-coated magnetic beads and a MACS separator (Miltenyi Biotec Inc.) following the manufacturer’s protocols as previously described [12,84]. The purity of isolated populations was monitored by FACS analysis, and cell preparations of >95% purity were used for RNA and protein extraction. Purified B cells were cultured *ex vivo* in the absence or presence of B cell stimuli for 6 or 24 hours as described previously [12,84]. B cell stimuli examined include 2 μg/ml anti-CD40, 20 μg/ml LPS, 1 μg/ml anti-BCR, and 100 nM CpG2084, alone or in combination. Total cellular RNA was prepared at 6 or 24 hours after stimulation.

### Taqman copy number assay of the mouse *MCC* gene

Genomic DNA was prepared from splenocytes of LMC and tumor-bearing B-TRAF3^−/−^ mice as previously described [13]. Quantitative real-time PCR of the mouse *MCC* gene was performed using the TaqMan Copy Number Assay kit (assay ID: Mm00490037_cn; Applied Biosystems) following the manufacturer’s protocols. Briefly, real-time PCR was performed using the TaqMan primers and probe (FAM-labeled) specific for the mouse MCC gene. Each reaction also included the probe (VIC-labeled) and primers specific for the mouse Trfc gene (TaqMan Copy Number Reference Assay, Applied Biosystems), which served as reference control. Relative copy numbers of the MCC gene in genomic DNA samples were analyzed using the Sequencing Detection Software (Applied Biosystems) and the comparative Ct method (ΔΔCt) following the manufacturer’s protocols.

### Chromatin immunoprecipitation assay

Chromatin immunoprecipitation (ChIP) assays were performed essentially as described previously [85,86]. Briefly, 20 × 10^6^ cell equivalents of fragmented chromatins (containing DNA fragments < 400 bp) were immunoprecipitated with Ig isotype control or antibodies specific for histone modifications. We used the following histone antibodies in the ChIP experiments: H3K27me3 (Millipore, Billerica, MA) and H3K9/14ac (Diagenode, Denville, NJ). Immunoprecipitated DNA were purified, and then analyzed by quantitative real time PCR using primers specific for the promoter region of the mouse *MCC* or *Diras2* gene. Primer used are: mMCC-U519F (5’- CAG GGA GGT TGG AGA GGA -3’) and mMCC-U446R (5’- AAA CAT GCC CTG CCC TTG -3’); mDiras2-U559F (5’- GCA CAT GTG ACT ACT ATT G -3’) and mDiras2-U480R (5’- AAT CTC TCC TCC CAC AAG -3’). The enrichments of each gene promoter immunoprecipitated by histone marks were quantitated relative to the input DNA [86].

### Cloning of the full-length cDNA of the *MCC* gene from TRAF3^−/−^ mouse B lymphomas and human MM cell lines

Total cellular RNA was prepared from B lymphomas spontaneously developed in four individual B-TRAF3^−/−^ mice (mouse ID: 6983–2, 7060–8, 105–8, and 115–6), and the corresponding cDNA samples were used as templates to clone the mouse MCC coding sequences using primers mMCC-F (5’- ATG AAT TCT GGA GTT GCG GTG -3’) and mMCC-R (5’- TTA GAG TGA CGT TTC GTT GGT G -3’). Similarly, human MCC coding sequences were cloned from human MM cell lines LP1 and KMS11 cells using reverse transcription PCR. Primers used for the cloning of human MCC are hMCC-F (5’- TGC ATC ATG AAT TCC GGA GT -3’), and hMCC-R (5’- TTA AAG CGA AGT TTC ATT GGT GTG -3’). The high fidelity polymerase Pfu UltraII (Santa Clara, CA) was used in these PCR reactions. Sequences of the cloned mouse and human MCC were determined at GenScript (Piscataway, NJ).

### Generation of lentiviral MCC expression and shRNA vectors

The coding cDNA sequence of *MCC* cloned from the human MM cell line LP1 cells was subcloned into the lentiviral expression vector pUB-eGFP-Thy1.1 [87] (generously provided by Dr. Zhibin Chen, the University of Miami, Miami, FL) by replacing the eGFP coding sequence with the MCC coding sequence. To facilitate immunoprecipitation experiments, we engineered an N-terminal FLAG tag or a C-terminal SBP-6×His tag [88] in frame with the MCC coding sequence, respectively. We subsequently generated two lentiviral expression vectors of tagged hMCC, including pUB-FLAG-hMCC and pUB-hMCC-SBP-6×His. Lentiviral shRNA vectors specific for human *MCC* (including hMCC shRNA 1332, 1388, 2284 and 2689; all in Torc1 vectors) or a scrambled shRNA vector were purchased from Sigma. To facilitate FACS analysis and cell sorting, we engineered an eGFP-expressing version of all the shRNA vectors by replacing the puromycin resistance gene of Torc1 with the eGFP coding sequence. Each lentiviral expression or shRNA vector was verified by DNA sequencing.

### Lentiviral packaging and transduction of human MM cells

Lentiviruses of MCC shRNA vectors and a scrambled shRNA vector were packaged following the manufacturer’s protocol (Sigma) as previously described [13,89]. Lentiviruses of MCC expression vectors were packaged and titered as previously described [67,80,87]. Human MM cells were transduced with the packaged lentiviruses at a MOI of 1:5 (cell:virus) in the presence of 8 μg/ml polybrene [13,87,89]. Transduction efficiency of cells was analyzed by flow cytometry on day 3 post transduction. All shRNA vectors contain an eGFP expression cassette, and were directly analyzed using a flow cytometer. All pUB lentiviral expression vectors have an expression cassette of the marker Thy1.1, and thus allow the transduced cells to be analyzed by Thy1.1 immunofluorescence staining followed by flow cytometry. Transduced cells were subsequently sorted or directly analyzed for apoptosis, cell cycle distribution, proliferation, and protein expression.

### Growth curve determination, annexin V staining of apoptotic cells, cell cycle distribution, and cell proliferation analyses

For MCC knockdown studies, on day 4 post-transduction, successfully transduced cells were sorted for GFP+ shRNA expressing populations, and then plated in 6-well plates for growth curve determination, annexin V staining, or cell cycle analysis. For growth curve determination, live and dead cells were differentiated using trypan blue staining, and counted using a hemacytometer. For analysis of apoptosis, cells were stained with annexin V and PI according to the manufacturer’s protocol (Invitrogen), and analyzed by flow cytometry as previously described [13]. For cell cycle analysis, cells were fixed with ice-cold 70% ethanol. Cell cycle distribution was subsequently determined by propidium iodide (PI) staining followed by flow cytometry as previously described [12,90]. For cell proliferation analysis, cells were labeled with a cell proliferation dye eFluor® 670 (eBioscience), and dilution of the proliferation dye was analyzed by flow cytometry following the manufacturer’s protocol.

### Total protein lysates, fractionation of cytosol, mitochondria and microsomes (rich in ER), and immunoblot analysis

For total protein lysates, cell pellets were lysed in 200 μl of 2X SDS sample buffer (0.0625 M Tris, pH6.8, 1% SDS, 15% glycerol, 2% β-mercaptoethanol and 0.005% bromophenol blue), sonicated for 30 pulses, and boiled for 10 minutes.

For biochemical fractionation, human MM cells (30 × 10^6^ cells/condition) were cultured in the absence or presence of 250 μM DTT or 0.5 μM thapsigargin for 24 hours. Cytosol, mitochondria and microsomes (rich in ER) were fractionated from cells as previously described [91–93]. Briefly, cells were washed with ice-cold PBS, swelled in 700 μl of Mitochondria Isolation Buffer (250 mM sucrose, 10 mM HEPES, pH7.5, 10 mM KCl, 1 mM EDTA, and 0.1 mM EGTA with protease and phosphatase inhibitors) on ice for 10 minutes, and then homogenized in a Dounce homogenizer. Cell lysis was checked by trypan blue uptake, and homogenization stopped when 90% of cells were broken. Nuclei were pelleted by centrifugation at 1,000 g for 10 minutes at 4°C. The cleared lysates were then centrifuged at 10,000 g for 25 minutes at 4°C to obtain the pellets of mitochondria. The supernatants were further centrifuged at 100,000 g for 2 hours to separate the pellets of microsomes (rich in ER) from cytosolic proteins (S100 fraction). One-fifth volume of 5X SDS sample buffer was added into each S100 fraction. The pellets of nuclei, mitochondria and microsomes (rich in ER) were lysed and sonicated in 300 μl of 2X SDS sample buffer, respectively. All protein samples were subsequently boiled for 10 minutes.

Total protein lysates, or cytosolic, ER, mitochondrial and nuclear proteins were separated by SDS-PAGE. Immunoblot analyses were performed using specific antibodies as previously described [67,80]. Images of immunoblots were acquired using a low-light imaging system (LAS-4000 mini, FUJIFILM Medical Systems USA, Inc., Stamford, CT).

### Co-immunoprecipitation assay in whole cell lysates

Human MM cell line 8226 cells (5 × 10^7^ cells/condition) transduced with pUB-FLAG-hMCC or pUB-hMCC-SBP-6×His were lysed and sonicated in the CHAPS lysis buffer [94] (1% CHAPS, 20 mM Tris, pH 7.4, 150 mM NaCl, 50 mM β-glycerophosphate, and 5% glycerol with freshly added 1 mM DTT and EDTA-free Mini-complete protease inhibitor cocktail). The insoluble pellets were removed by centrifugation at 10,000 g for 20 minutes at 4°C. The CHAPS lysates were subsequently immunoprecipitated with anti-FLAG-agarose beads (Sigma; for FLAG-hMCC), or streptavidin-sepharose beads (Pierce, Rockford, IL; for hMCC-SBP-6×His), separately. Immunoprecipitates were washed 5 times with the Wash Buffer (0.5% CHAPS, 20 mM Tris, pH 7.4, 150 mM NaCl, 50 mM β-glycerophosphate, and 2.5% glycerol with freshly added 1 mM DTT and EDTA-free Mini-complete protease inhibitor cocktail). Immunoprecipitated proteins were resuspended in 2× SDS sample buffer, boiled for 10 minutes, and then separated on SDS-PAGE for mass spectrometry or immunoblot analyses.

### Co-immunoprecipitation assay in mitochondrial lysates

Human MM cell line 8226 cells (1.5 × 10^8^ cells/condition) transduced with pUB-FLAG-hMCC or pUB-hMCC-SBP-6×His were used for cytosol, mitochondria and ER fractionation as described above. Mitochondrial pellets were lysed and sonicated in the CHAPS lysis buffer [94], and cleared by centrifugation at 10,000 g for 20 minutes at 4°C. The mitochondrial lysates were subsequently immunoprecipitated with streptavidin-sepharose beads (Pierce, Rockford, IL; for hMCC-SBP-6×His). Immunoprecipitates were washed 5 times with the Wash Buffer, resuspended in 2× SDS sample buffer, boiled for 10 minutes, and then separated on SDS-PAGE for mass spectrometry or immunoblot analyses.

### Mass spectrometry based-sequencing

Whole cell lysates or mitochondrial lysates immunoprecipitated with streptavidin-sepharose beads were used for LC-MS/MS. The entire gel lanes for hMCC-SBP-6×His complex and negative control (FLAG-hMCC) were each sectioned into 15 continuous slices. The gel slice samples were subjected to thiol reduction by TCEP, alkylation with iodoacetamide, and digestion with sequencing-grade modified trypsin [95,96]. Peptides were eluted from the gel slices, desalted, and then subjected to reversed-phase nano-flow ultra high performance capillary liquid chromatography (uPLC) followed by high-resolution/high-mass accuracy MS/MS analysis using an LC-MS platform consisting of an Eksigent Nano Ultra 2D Plus uPLC system hyphenated to a Thermo Orbi Velos mass spectrometer. The MS/MS was set to operate in data dependent acquisition mode using a duty cycle in which the top 15 most abundant peptide ions in the full scan MS were targeted for MS/MS sequencing. Full scan MS1 spectra were acquired at 100,000 resolving power and maintained mass calibration to within 2–3 ppm mass accuracy. LC-MS/MS data were searched against the human IPI and UniProt databases using the Mascot and Proteome Discoverer search engines [95,96]. Protein assignments were considered highly confident using a stringent false discovery rate threshold of <1%, as estimated by reversed database searching, and requiring that ≥2 peptides per protein be unambiguously identified. Rough relative protein amounts were estimated using spectra counting values.

### Statistics

Statistical analyses were performed using the Prism software (GraphPad, La Jolla, CA). Statistical significance was assessed by Student *t* test. *P* values less than 0.05 are considered significant.

## Additional files

Additional file 1: Table S1. List of genes differentially expressed in TRAF3^-/-^ mouse splenic B lymphomas identified by the microarray analysis. Additional file 2: Table S2. The MCC-interactome in human MM cells identified by affinity purification followed by LC-MS/MS. Additional file 3: Figure S1. The MCC isoform 2 was identified as an MCC-interacting protein by affinity purification and LC-MS/MS. The two isoforms of MCC proteins differ at their extreme N-terminus due to alternative promoter usage. Both pUB-FLAG-hMCC and pUB-hMCC-SBP-6xHis are cloned from MCC isoform 1 (829 aa). **(A)** Schematic diagram of peptide sequences of MCC isoform 1 identified by LC-MS/MS. **(B)** Schematic diagram of peptide sequences of MCC isoform 2 (1019 aa) identified by LC-MS/MS. The peptide sequences detected by LC-MS/MS are highlighted in green color. The unique region of MCC isofrom 2 is marked with a dashed black box.

## Abbreviations

MCC: Mutated in colorectal cancer
TRAF3: Tumor necrosis factor receptor (TNF-R)-associated factor 3
B-TRAF3^−/−^: B cell-specific TRAF3-deficient
NHL: Non-Hodgkin lymphoma
MM: Multiple myeloma
MZL: Splenic marginal zone lymphoma
B-CLL: B cell chronic lymphocytic leukemia
MCL: Mantle cell lymphoma
WM: Waldenström’s macroglobulinemia
LOH: Loss of heterozygosity
FAP: Familial adenomatous polyposis
CRCs: Colorectal cancers
PEL: Primary effusion lymphoma
CBL: Centroblastic lymphoma
DLBCL: Diffuse large B-cell lymphoma
BL: Burkitt’s lymphoma
NF-κB: Nuclear factor κ light chain enhancer of activated B cells
BCR: B cell receptor
LPS: Lipopolysaccharides
shRNA: Short hairpin RNA
ChIP: Chromatin immunoprecipitation
H3K27Me3: trimethylated lysine 27 of histone 3
H3K9/14Ac: Acetylated lysine 9/14 of histone 3
DTT: Dithiothreitol
ERK: Extracellular signal-regulated kinase
JNK: C-Jun N-terminal kinase
SBP: Streptavidin-binding peptide
SA: Streptavidin
LC-MS/MS: Liquid chromatography-mass spectrometry/mass spectrometry
uPLC: High performance capillary liquid chromatography
PARP1: Poly [ADP-ribose] polymerase 1
PHB2: Prohibitin-2
PHB: Prohibitin
PI: Propidium iodide
FACS: Fluorescence-activated cell sorting
PCR: Polymerase chain reaction
