# Supplementary material for: Mutated in colorectal cancer (MCC) is a novel oncogene in B lymphocytes
Source: J Hematol Oncol. 2014 Sep 9;7:56. doi: 10.1186/s13045-014-0056-6 (PMC4172902; doi:10.1186/s13045-014-0056-6)
Supplement: Additional file 1: Table S1. — List of genes differentially expressed in TRAF3-/- mouse splenic B lymphomas identified by the microarray analysis. [file 13045_2014_56_MOESM1_ESM.pdf]

Supplementary Table 1. List of genes differentially expressed in TRAF3$^{-/-}$ mouse splenic B lymphomas identified by the microarray analysis

#	GeneSymbol	Gene Name	LogFC	AveExpr	t	P. Value	Adj. P. Val.
1	Zcwpw1	zinc finger, CW type with PWWP domain 1	3.36	9.50	14.20	4.07E-07	0.000126365
2	Diras2	DIRAS family, GTP-binding RAS-like 2	3.35	10.56	14.57	3.32E-07	0.000126365
3	Serpina3f	serine (or cysteine) peptidase inhibitor, clade A, member 3F	3.03	10.87	9.99	6.54E-06	0.000571
4	Sox5	SRY-box containing gene 5	2.93	9.48	13.97	4.65E-07	0.000130244
5	C130026I21Rik	RIKEN cDNA C130026I21 gene	2.87	9.95	5.58	0.000456113	0.008256021
6	Tnfrsf19	tumor necrosis factor receptor superfamily, member 19	2.87	9.00	13.59	5.78E-07	0.000148597
7	Mcc	mutated in colorectal cancers	2.76	8.68	31.01	6.98E-10	9.15E-06
8	Slamf9	SLAM family member 9	2.75	10.73	4.75	0.00131062	0.016097166
9	Fah	fumarylacetoacetate hydrolase	2.63	11.18	7.83	4.13E-05	0.00178595
10	Rdh12	retinol dehydrogenase 12	2.61	11.29	12.81	9.27E-07	0.000205961
11	Ahnak2	AHNAK nucleoprotein 2	2.54	8.93	12.49	1.13E-06	0.000226433
12	Chst7	carbohydrate (N-acetylglucosamino) sulfotransferase 7	2.42	9.23	7.85	4.05E-05	0.001759595
13	Vars	valyl-tRNA synthetase	2.30	11.46	16.48	1.23E-07	8.74E-05
14	Twsg1	twisted gastrulation homolog 1 (Drosophila)	2.30	10.96	10.51	4.41E-06	0.000441575
15	Tbc1d9	TBC1 domain family, member 9	2.29	9.53	17.05	9.34E-08	7.20E-05
16	Vars	valyl-tRNA synthetase	2.13	9.29	17.17	8.83E-08	7.20E-05
17	Cd59a	CD59a antigen	2.11	10.04	5.72	0.000385181	0.007336917
18	Gnb3	guanine nucleotide binding protein (G protein), beta 3	2.09	8.73	5.99	0.000283427	0.006099042
19	Sspn	sarcospan	2.08	10.14	4.98	0.000958982	0.013242851
20	Lacc1	laccase (multicopper oxidoreductase) domain containing 1	2.08	8.98	19.86	2.70E-08	5.06E-05
21	Gbp1	guanylate binding protein 1	2.06	9.78	4.19	0.00279695	0.02699119
22	Vars	valyl-tRNA synthetase	2.06	12.05	21.24	1.56E-08	3.41E-05
23	Ccbp2	chemokine binding protein 2	1.96	9.96	7.62	5.07E-05	0.002000536
24	Gng13	guanine nucleotide binding protein (G protein), gamma 13	1.94	8.60	7.33	6.70E-05	0.00236437
25	Plscr1	phospholipid scramblase 1	1.94	10.35	6.13	0.000239207	0.005519038
26	Clic4	chloride intracellular channel 4 (mitochondrial)	1.93	12.48	7.57	5.33E-05	0.002054453
27	Sema7a	sema domain, immunoglobulin domain (Ig), and GPI membrane anchor, (sema	1.91	9.22	9.70	8.22E-06	0.000643485
28	Chrnb1	cholinergic receptor, nicotinic, beta polypeptide 1 (muscle)	1.89	9.84	8.35	2.55E-05	0.001291342
29	Ppap2b	phosphatidic acid phosphatase type 2B	1.86	10.46	18.00	6.01E-08	7.20E-05
30	Ebi3	Epstein-Barr virus induced gene 3	1.82	9.85	10.82	3.51E-06	0.00039538
31	4930539E08Rik	RIKEN cDNA 4930539E08 gene	1.81	8.92	8.10	3.22E-05	0.001536721
32	Nacc2	nucleus accumbens associated 2, BEN and BTB (POZ) domain containing	1.79	9.23	12.76	9.57E-07	0.000209017
33	Fcrl5	Fc receptor-like 5	1.79	9.62	8.79	1.74E-05	0.001046831
34	Sel1l3	sel-1 suppressor of lin-12-like 3 (C. elegans)	1.76	8.30	7.70	4.70E-05	0.001893452
35	Dnajc7	DnaJ (Hsp40) homolog, subfamily C, member 7	1.76	10.73	6.73	0.000124642	0.003589973
36	Pdlim1	PDZ and LIM domain 1 (elfin)	1.74	11.75	6.79	0.000116482	0.003392213
37	Abca3	ATP-binding cassette, sub-family A (ABC1), member 3	1.71	10.86	11.21	2.66E-06	0.0003633
38	Rassf4	Ras association (RalGDS/AF-6) domain family member 4	1.66	11.54	10.68	3.89E-06	0.000397578
39	Kynu	kynureninase (L-kynurenine hydrolase)	1.65	10.56	14.43	3.57E-07	0.000126365
40	Cd274	CD274 antigen	1.64	11.38	16.17	1.43E-07	8.74E-05

41	Cd80	CD80 antigen	1.64	8.83	12.33	1.25E-06	0.00023571
42	Ccdc28b	coiled coil domain containing 28B	1.64	10.34	7.19	7.74E-05	0.002628686
43	Rassf4	Ras association (RalGDS/AF-6) domain family member 4	1.63	11.53	11.04	3.00E-06	0.000376611
44	Sp140	Sp140 nuclear body protein	1.63	9.80	11.51	2.15E-06	0.000322971
45	AF067061	cDNA sequence AF067061	1.63	8.54	4.77	0.001275097	0.015838995
46	Ero1lb	ERO1-like beta (S. cerevisiae)	1.63	11.22	12.45	1.16E-06	0.000226433
47	Nfatc1	nuclear factor of activated T cells, cytoplasmic, calcineurin dependent 1	1.61	9.89	8.27	2.76E-05	0.001378762
48	Hdac4	histone deacetylase 4	1.61	8.78	8.11	3.17E-05	0.001523759
49	Tubb2b	tubulin, beta 2B class IIB	1.60	10.65	5.22	0.000712545	0.010897018
50	Trim40	tripartite motif-containing 40	1.59	8.56	8.79	1.74E-05	0.001046831
51	Plscr1	phospholipid scramblase 1	1.57	9.33	6.70	0.000129091	0.003645988
52	Arhgap24	Rho GTPase activating protein 24	1.52	10.13	11.91	1.65E-06	0.000270244
53	D13Ertd608e	DNA segment, Chr 13, ERATO Doi 608, expressed	1.52	8.97	10.25	5.35E-06	0.0005051
54	Hn1l	hematological and neurological expressed 1-like	1.52	9.27	15.10	2.49E-07	0.000112734
55	Ccdc28b	coiled coil domain containing 28B	1.50	9.62	9.68	8.31E-06	0.000643485
56	Tcf4	transcription factor 4	1.50	12.04	9.19	1.24E-05	0.000847921
57	Zbtb32	zinc finger and BTB domain containing 32	1.49	8.54	9.39	1.05E-05	0.000754882
58	Gbp2	guanylate binding protein 2	1.48	11.56	7.68	4.79E-05	0.001904754
59	Caln1	calneuron 1	1.48	8.43	6.18	0.000228083	0.005366295
60	Gpr34	G protein-coupled receptor 34	1.48	8.77	6.37	0.000182843	0.004607992
61	Serpina3g	serine (or cysteine) peptidase inhibitor, clade A, member 3G	1.47	12.85	8.47	2.30E-05	0.001206517
62	Rbm47	RNA binding motif protein 47	1.45	9.83	11.02	3.04E-06	0.000376611
63	Fgd2	FYVE, RhoGEF and PH domain containing 2	1.45	11.91	5.76	0.000368771	0.007141322
64	Fgd6	FYVE, RhoGEF and PH domain containing 6	1.44	9.42	8.42	2.41E-05	0.001236456
65	Neurod4	neurogenic differentiation 4	1.43	8.19	7.07	8.74E-05	0.002845238
66	Rassf4	Ras association (RalGDS/AF-6) domain family member 4	1.42	10.14	7.45	5.94E-05	0.002191762
67	Rhbdf1	rhomboid family 1 (Drosophila)	1.42	10.30	8.74	1.81E-05	0.001056366
68	Gstt3	glutathione S-transferase, theta 3	1.40	9.14	6.47	0.000164429	0.004282628
69	Pafah1b3	platelet-activating factor acetylhydrolase, isoform 1b, subunit 3	1.40	10.94	5.50	0.000501117	0.00873186
70	Asph	aspartate-beta-hydroxylase	1.37	8.04	5.64	0.000426167	0.007789294
71	Nid1	nidogen 1	1.37	8.57	7.77	4.37E-05	0.001817842
72	Sgk3	serum/glucocorticoid regulated kinase 3	1.36	9.50	10.11	5.97E-06	0.000539159
73	Gas7	growth arrest specific 7	1.35	8.81	6.41	0.000176165	0.004524439
74	NA	NA	1.34	10.20	11.26	2.56E-06	0.000352915
75	Gm14137	predicted gene 14137	1.34	8.18	17.51	7.51E-08	7.20E-05
76	Prps2	phosphoribosyl pyrophosphate synthetase 2	1.33	9.69	8.55	2.13E-05	0.001175007
77	Sox5	SRY-box containing gene 5	1.32	8.42	11.10	2.87E-06	0.000365287
78	Hsp90ab1	heat shock protein 90 alpha (cytosolic), class B member 1	1.30	11.59	7.12	8.32E-05	0.002740232
79	Eps15	epidermal growth factor receptor pathway substrate 15	1.30	9.95	9.51	9.50E-06	0.000711445
80	Cybasc3	cytochrome b, ascorbate dependent 3	1.29	10.90	11.68	1.92E-06	0.000303211
81	Fbxw13	F-box and WD-40 domain protein 13	1.29	8.28	9.97	6.61E-06	0.000572834
82	Recql5	RecQ protein-like 5	1.28	9.46	7.40	6.26E-05	0.002267958
83	Kcnk5	potassium channel, subfamily K, member 5	1.28	10.16	10.71	3.81E-06	0.00039567
84	Ptpn22	protein tyrosine phosphatase, non-receptor type 22 (lymphoid)	1.27	11.57	8.75	1.80E-05	0.001056366

85	Pdia4	protein disulfide isomerase associated 4	1.27	12.33	7.50	5.68E-05	0.002143803
86	Ccnd2	cyclin D2	1.27	10.35	7.56	5.37E-05	0.002064785
87	Plac1l	placenta-specific 1-like	1.27	8.63	7.49	5.74E-05	0.002156914
88	Gpr137b	G protein-coupled receptor 137B	1.26	8.68	8.07	3.31E-05	0.001539334
89	R74862	expressed sequence R74862	1.26	8.57	8.55	2.13E-05	0.001175007
90	Cfp	complement factor properdin	1.25	13.35	8.51	2.22E-05	0.001185616
91	Plp2	proteolipid protein 2	1.24	10.88	5.00	0.000937488	0.013156142
92	Blm	Bloom syndrome, RecQ helicase-like	1.23	10.01	10.89	3.33E-06	0.000389533
93	Slc29a3	solute carrier family 29 (nucleoside transporters), member 3	1.23	9.84	11.50	2.17E-06	0.000322971
94	Neo1	neogenin	1.23	8.29	5.97	0.00028734	0.006114237
95	Gpr34	G protein-coupled receptor 34	1.23	8.84	3.81	0.004793627	0.038587517
96	Lysmd2	LysM, putative peptidoglycan-binding, domain containing 2	1.22	8.80	9.68	8.32E-06	0.000643485
97	Optn	optineurin	1.22	8.75	8.54	2.16E-05	0.001177646
98	D10Wsu102e	DNA segment, Chr 10, Wayne State University 102, expressed	1.22	9.14	10.67	3.91E-06	0.000397578
99	Igsf9	immunoglobulin superfamily, member 9	1.22	8.76	8.60	2.06E-05	0.001143744
100	Hmgn3	high mobility group nucleosomal binding domain 3	1.21	11.16	4.63	0.001537694	0.018154491
101	Man1a	mannosidase 1, alpha	1.21	9.81	7.55	5.42E-05	0.002069558
102	Robo1	roundabout homolog 1 (Drosophila)	1.21	8.23	5.95	0.000295706	0.006190469
103	Hmgn3	high mobility group nucleosomal binding domain 3	1.21	11.28	3.69	0.005709615	0.043452096
104	Apoe	apolipoprotein E	1.20	9.14	5.38	0.000582464	0.009505843
105	Oosp1	oocyte secreted protein 1	1.20	10.03	3.57	0.006900257	0.049987767
106	Nsf	N-ethylmaleimide sensitive fusion protein	1.20	10.71	10.25	5.36E-06	0.0005051
107	Rap1gap2	RAP1 GTPase activating protein 2	1.19	9.90	5.20	0.000724814	0.011044987
108	Camk2d	calcium/calmodulin-dependent protein kinase II, delta	1.19	9.22	8.84	1.66E-05	0.001025724
109	Il10	interleukin 10	1.19	8.19	11.29	2.51E-06	0.000352016
110	Ticam2	toll-like receptor adaptor molecule 2	1.19	8.59	14.17	4.14E-07	0.000126365
111	NA	NA	1.18	8.92	7.95	3.69E-05	0.001646747
112	Sgk3	serum/glucocorticoid regulated kinase 3	1.18	9.77	14.40	3.65E-07	0.000126365
113	Tcstv1	2-cell-stage, variable group, member 1	1.18	8.27	4.56	0.001684377	0.019177893
114	Rilpl2	Rab interacting lysosomal protein-like 2	1.18	10.26	4.87	0.001106368	0.014432138
115	Dnase1l3	deoxyribonuclease 1-like 3	1.17	9.18	7.37	6.46E-05	0.002318592
116	Suclg2	succinate-Coenzyme A ligase, GDP-forming, beta subunit	1.17	10.23	3.86	0.004475495	0.036934103
117	NA	NA	1.17	9.63	4.38	0.002134617	0.022487262
118	Ivns1abp	influenza virus NS1A binding protein	1.16	10.99	5.23	0.000701269	0.010805477
119	Rtn4ip1	reticulon 4 interacting protein 1	1.16	8.44	8.02	3.46E-05	0.001575493
120	Gpm6a	glycoprotein m6a	1.16	8.19	13.33	6.77E-07	0.000167465
121	Tmem154	transmembrane protein 154	1.15	10.03	16.07	1.50E-07	8.74E-05
122	Cd59b	CD59b antigen	1.15	8.70	5.23	0.0007025	0.010805477
123	NA	NA	1.14	8.20	11.11	2.86E-06	0.000365287
124	Tbc1d5	TBC1 domain family, member 5	1.13	10.54	7.82	4.17E-05	0.00179651
125	Lrrc8a	leucine rich repeat containing 8A	1.13	8.66	5.80	0.000350171	0.006880043
126	Optn	optineurin	1.12	8.65	7.60	5.16E-05	0.002011249
127	Timd2	T cell immunoglobulin and mucin domain containing 2	1.12	8.33	8.74	1.82E-05	0.001056366
128	Cobll1	Cobl-like 1	1.11	9.58	7.97	3.61E-05	0.001626306

129	Gbp3	guanylate binding protein 3	1.10	11.00	7.46	5.93E-05	0.002191762
130	Ftsjd2	FtsJ methyltransferase domain containing 2	1.10	12.78	7.16	7.94E-05	0.002664622
131	Kctd12	potassium channel tetramerisation domain containing 12	1.10	9.91	7.79	4.30E-05	0.00181213
132	Chrnb1	cholinergic receptor, nicotinic, beta polypeptide 1 (muscle)	1.09	8.73	7.03	9.11E-05	0.002946967
133	Psmd14	proteasome (prosome, macropain) 26S subunit, non-ATPase, 14	1.09	10.25	8.53	2.17E-05	0.001177646
134	Irak2	interleukin-1 receptor-associated kinase 2	1.09	10.08	12.03	1.52E-06	0.000258979
135	Fcho2	FCH domain only 2	1.08	11.25	9.05	1.40E-05	0.000906822
136	Tnfsf4	tumor necrosis factor (ligand) superfamily, member 4	1.07	8.38	5.71	0.000389426	0.007374903
137	Hsp90ab1	heat shock protein 90 alpha (cytosolic), class B member 1	1.07	11.52	6.39	0.000179522	0.004550005
138	Fut8	fucosyltransferase 8	1.06	8.78	7.94	3.74E-05	0.001653635
139	Ryk	receptor-like tyrosine kinase	1.06	9.24	7.63	5.01E-05	0.001984949
140	Myadm	myeloid-associated differentiation marker	1.05	10.34	6.12	0.000242092	0.005562299
141	Tgif1	TGFB-induced factor homeobox 1	1.05	9.59	7.81	4.20E-05	0.001800796
142	Cobll1	Cobl-like 1	1.05	9.11	10.81	3.53E-06	0.00039538
143	Il15	interleukin 15	1.05	8.97	3.89	0.004302602	0.036006133
144	Ints4	integrator complex subunit 4	1.04	11.45	13.91	4.82E-07	0.000130244
145	Camk2n1	calcium/calmodulin-dependent protein kinase II inhibitor 1	1.04	8.74	4.50	0.001832528	0.02028317
146	Fert2	fer (fms/fps related) protein kinase, testis specific 2	1.04	8.04	13.68	5.50E-07	0.000144109
147	Gbp2	guanylate binding protein 2	1.03	8.47	7.78	4.32E-05	0.00181213
148	Aifm2	apoptosis-inducing factor, mitochondrion-associated 2	1.03	8.85	8.25	2.80E-05	0.00138606
149	F9	coagulation factor IX	1.03	8.67	9.68	8.30E-06	0.000643485
150	Ppfibp2	PTPRF interacting protein, binding protein 2 (liprin beta 2)	1.03	8.92	7.87	3.98E-05	0.001737923
151	Marcks	myristoylated alanine rich protein kinase C substrate	1.03	11.22	6.39	0.000179412	0.004550005
152	Sh3bgrl2	SH3 domain binding glutamic acid-rich protein like 2	1.03	8.48	4.36	0.002212342	0.023065033
153	Rab31	RAB31, member RAS oncogene family	1.02	10.80	4.78	0.001244962	0.015627613
154	Unc119	unc-119 homolog (C. elegans)	1.02	9.44	5.65	0.000422166	0.007726939
155	Cbfa2t3	core-binding factor, runt domain, alpha subunit 2, translocated to, 3 (human)	1.01	9.74	6.72	0.000125996	0.003600003
156	Ly96	lymphocyte antigen 96	1.01	9.80	5.08	0.000853268	0.012383256
157	NA	NA	1.01	10.56	8.60	2.06E-05	0.001143744
158	Irak2	interleukin-1 receptor-associated kinase 2	1.01	9.42	12.27	1.30E-06	0.000236954
159	Cyp51	cytochrome P450, family 51	1.01	9.19	5.56	0.000465437	0.008365629
160	Cyb5r2	cytochrome b5 reductase 2	1.00	8.00	4.16	0.002895417	0.027587193
161	Tmem38b	transmembrane protein 38B	-1.00	8.78	-8.49	2.25E-05	0.001195989
162	Sit1	suppression inducing transmembrane adaptor 1	-1.01	8.65	-8.52	2.20E-05	0.001183798
163	Fam117a	family with sequence similarity 117, member A	-1.02	10.61	-4.07	0.003303844	0.030200374
164	Abhd8	abhydrolase domain containing 8	-1.02	8.87	-9.44	1.01E-05	0.00074318
165	Rgcc	regulator of cell cycle	-1.02	9.66	-4.43	0.001991212	0.021459569
166	P2ry6	pyrimidinergic receptor P2Y, G-protein coupled, 6	-1.03	8.68	-6.88	0.000106007	0.003202773
167	Cyp27a1	cytochrome P450, family 27, subfamily a, polypeptide 1	-1.03	8.45	-6.08	0.000253691	0.005670363
168	Fcgrt	Fc receptor, IgG, alpha chain transporter	-1.03	9.14	-4.21	0.002699974	0.026333605
169	Rnf144a	ring finger protein 144A	-1.03	8.65	-6.75	0.000121862	0.003541023
170	Gm5483	predicted gene 5483	-1.04	8.51	-4.12	0.003099077	0.028885777
171	Gata3	GATA binding protein 3	-1.04	8.38	-12.33	1.26E-06	0.00023571
172	Ddit4	DNA-damage-inducible transcript 4	-1.04	10.01	-5.42	0.000556829	0.009225337

173	Mgst1	microsomal glutathione S-transferase 1	-1.05	8.54	-3.57	0.006872263	0.049887542
174	Nrp1	neuropilin 1	-1.05	8.57	-6.12	0.000243368	0.005575765
175	Frat2	frequently rearranged in advanced T cell lymphomas 2	-1.05	8.43	-11.89	1.67E-06	0.000270244
176	Brwd1	bromodomain and WD repeat domain containing 1	-1.05	9.29	-3.86	0.004465454	0.036920991
177	P2ry14	purinergic receptor P2Y, G-protein coupled, 14	-1.05	8.76	-9.39	1.05E-05	0.000754882
178	Gmfg	glia maturation factor, gamma	-1.06	11.65	-4.91	0.001051831	0.013994156
179	Akap12	A kinase (PRKA) anchor protein (gravin) 12	-1.06	8.18	-7.97	3.64E-05	0.001633462
180	Gpr83	G protein-coupled receptor 83	-1.06	8.47	-9.72	8.06E-06	0.000643485
181	Cd27	CD27 antigen	-1.06	8.66	-12.95	8.50E-07	0.000191956
182	Apol7c	apolipoprotein L 7c	-1.06	8.64	-6.23	0.000214469	0.005176085
183	Socs3	suppressor of cytokine signaling 3	-1.06	10.41	-6.32	0.00019337	0.004772354
184	Serpinb6a	serine (or cysteine) peptidase inhibitor, clade B, member 6a	-1.07	9.46	-8.26	2.78E-05	0.00138606
185	Hpgd	hydroxyprostaglandin dehydrogenase 15 (NAD)	-1.07	8.13	-5.49	0.000510547	0.008795466
186	Xdh	xanthine dehydrogenase	-1.07	8.70	-9.00	1.45E-05	0.000923744
187	Xcl1	chemokine (C motif) ligand 1	-1.07	8.29	-13.93	4.75E-07	0.000130244
188	Zap70	zeta-chain (TCR) associated protein kinase	-1.08	8.76	-7.71	4.62E-05	0.001888593
189	Lrig1	leucine-rich repeats and immunoglobulin-like domains 1	-1.08	8.39	-10.76	3.66E-06	0.00039567
190	Klhl6	kelch-like 6	-1.08	12.72	-4.80	0.001218916	0.015444675
191	Pilrb1	paired immunoglobin-like type 2 receptor beta 1	-1.08	8.38	-9.71	8.15E-06	0.000643485
192	Klra7	killer cell lectin-like receptor, subfamily A, member 7	-1.09	8.24	-6.95	9.86E-05	0.003077668
193	Stat4	signal transducer and activator of transcription 4	-1.09	8.58	-7.78	4.35E-05	0.001817277
194	Nsg2	neuron specific gene family member 2	-1.09	8.21	-5.72	0.000388558	0.007374903
195	Sepp1	selenoprotein P, plasma, 1	-1.09	9.20	-7.46	5.92E-05	0.002191762
196	Prkcq	protein kinase C, theta	-1.09	8.51	-9.26	1.17E-05	0.000820031
197	Gstk1	glutathione S-transferase kappa 1	-1.09	8.52	-10.55	4.26E-06	0.000429447
198	Arrdc4	arrestin domain containing 4	-1.09	8.62	-9.97	6.64E-06	0.000572834
199	Stard10	START domain containing 10	-1.10	9.46	-5.06	0.000867359	0.012512813
200	Fam189b	family with sequence similarity 189, member B	-1.10	8.64	-5.67	0.000407926	0.007601808
201	Acss2	acyl-CoA synthetase short-chain family member 2	-1.10	9.26	-4.55	0.001709577	0.019280562
202	Tnfaip8l2	tumor necrosis factor, alpha-induced protein 8-like 2	-1.10	9.45	-14.09	4.34E-07	0.000126365
203	Rab3d	RAB3D, member RAS oncogene family	-1.10	9.20	-6.95	9.83E-05	0.003077254
204	Lpl	lipoprotein lipase	-1.10	8.47	-7.03	9.09E-05	0.002946967
205	Smad1	SMAD family member 1	-1.10	8.66	-6.20	0.000221278	0.005282699
206	Cdk5r1	cyclin-dependent kinase 5, regulatory subunit 1 (p35)	-1.11	9.32	-4.76	0.001283768	0.015886479
207	Apobr	apolipoprotein B receptor	-1.11	8.51	-6.82	0.00011301	0.003305794
208	Nrn1	neuritin 1	-1.11	8.48	-5.92	0.000306885	0.006333433
209	Cd14	CD14 antigen	-1.12	8.42	-7.59	5.21E-05	0.002014325
210	Clec4b1	C-type lectin domain family 4, member b1	-1.12	8.45	-7.25	7.28E-05	0.00250334
211	Smad1	SMAD family member 1	-1.13	8.63	-8.82	1.70E-05	0.001044514
212	Slc11a1	solute carrier family 11 (proton-coupled divalent metal ion transporters), membe	-1.13	8.94	-5.50	0.000504008	0.008759984
213	Prkd3	protein kinase D3	-1.13	8.79	-5.06	0.000872332	0.012548754
214	Krt10	keratin 10	-1.13	8.55	-14.30	3.85E-07	0.000126365
215	Vopp1	vesicular, overexpressed in cancer, prosurvival protein 1	-1.13	9.30	-5.65	0.000419539	0.007700368
216	Nkg7	natural killer cell group 7 sequence	-1.14	11.33	-4.16	0.002918159	0.027732032

217	Tcf7	transcription factor 7, T cell specific	-1.14	8.18	-5.88	0.000321124	0.00652455
218	Klk1	kallikrein 1	-1.14	8.12	-4.59	0.001608869	0.018579935
219	Hcst	hematopoietic cell signal transducer	-1.15	10.76	-5.30	0.00064044	0.010185633
220	Lck	lymphocyte protein tyrosine kinase	-1.15	11.14	-5.56	0.000467913	0.008365629
221	LOC547323	uncharacterized LOC547323	-1.15	8.57	-12.71	9.89E-07	0.000212413
222	NA	NA	-1.16	8.67	-10.00	6.49E-06	0.000571
223	Tmem66	transmembrane protein 66	-1.16	9.48	-7.35	6.56E-05	0.002342512
224	Pygl	liver glycogen phosphorylase	-1.17	9.54	-3.58	0.006723284	0.049085591
225	Cst3	cystatin C	-1.17	12.82	-6.51	0.000156878	0.004170149
226	Kmo	kynurenine 3-monooxygenase (kynurenine 3-hydroxylase)	-1.17	8.51	-7.15	8.07E-05	0.002684506
227	Ear10	eosinophil-associated, ribonuclease A family, member 10	-1.18	8.48	-7.67	4.80E-05	0.001904754
228	Hist1h1c	histone cluster 1, H1c	-1.18	10.21	-10.20	5.56E-06	0.000513086
229	Acpl2	acid phosphatase-like 2	-1.18	8.76	-7.89	3.91E-05	0.001713178
230	Pi16	peptidase inhibitor 16	-1.19	8.55	-6.92	0.000102002	0.003130533
231	NA	NA	-1.20	8.87	-15.10	2.49E-07	0.000112734
232	Prkd3	protein kinase D3	-1.21	9.48	-4.03	0.003499375	0.031290611
233	Nrp1	neuropilin 1	-1.21	8.85	-5.06	0.000867924	0.012512813
234	Anxa1	annexin A1	-1.21	8.46	-4.22	0.002662353	0.026134882
235	Il27ra	interleukin 27 receptor, alpha	-1.22	11.04	-9.74	7.94E-06	0.000643485
236	Selplg	selectin, platelet (p-selectin) ligand	-1.22	10.59	-7.42	6.13E-05	0.002226548
237	Lpar6	lysophosphatidic acid receptor 6	-1.22	10.65	-12.22	1.35E-06	0.000242138
238	Ppic	peptidylprolyl isomerase C	-1.23	8.33	-4.05	0.003389681	0.030741707
239	Sh2d2a	SH2 domain protein 2A	-1.23	10.75	-5.55	0.000476	0.008464011
240	Ear12	eosinophil-associated, ribonuclease A family, member 12	-1.23	8.60	-10.75	3.69E-06	0.00039567
241	Hvcn1	hydrogen voltage-gated channel 1	-1.24	12.02	-5.22	0.000712795	0.010897018
242	Sun2	Sad1 and UNC84 domain containing 2	-1.25	11.74	-10.93	3.24E-06	0.000384201
243	Fyb	FYN binding protein	-1.25	9.43	-9.69	8.24E-06	0.000643485
244	Fxyd5	FXYD domain-containing ion transport regulator 5	-1.25	10.04	-7.25	7.25E-05	0.00250152
245	Lrrk2	leucine-rich repeat kinase 2	-1.26	9.33	-3.86	0.004483801	0.036956105
246	Neurl3	neuralized homolog 3 homolog (Drosophila)	-1.27	8.57	-9.06	1.38E-05	0.000901477
247	NA	NA	-1.28	9.30	-14.09	4.34E-07	0.000126365
248	Sit1	suppression inducing transmembrane adaptor 1	-1.28	8.95	-10.25	5.34E-06	0.0005051
249	Foxp3	forkhead box P3	-1.28	8.88	-11.28	2.52E-06	0.000352016
250	Klf13	Kruppel-like factor 13	-1.29	10.80	-7.22	7.52E-05	0.002573373
251	Cd160	CD160 antigen	-1.29	8.72	-8.53	2.17E-05	0.001177646
252	Fas	Fas (TNF receptor superfamily member 6)	-1.29	8.97	-9.43	1.02E-05	0.00074318
253	Ifngr1	interferon gamma receptor 1	-1.29	9.89	-7.94	3.72E-05	0.001650647
254	Fam78a	family with sequence similarity 78, member A	-1.30	11.06	-6.07	0.000258382	0.005742531
255	Osm	oncostatin M	-1.30	9.04	-17.16	8.87E-08	7.20E-05
256	Asb2	ankyrin repeat and SOCS box-containing 2	-1.30	9.77	-7.78	4.33E-05	0.00181213
257	Tyrobp	TYRO protein tyrosine kinase binding protein	-1.31	9.68	-8.30	2.69E-05	0.001355341
258	Casp1	caspase 1	-1.32	11.79	-5.17	0.000758776	0.01139033
259	Bcl7a	B cell CLL/lymphoma 7A	-1.32	9.92	-9.88	7.12E-06	0.000602323
260	Cd247	CD247 antigen	-1.32	9.05	-12.17	1.39E-06	0.0002457

261	Zfp36	zinc finger protein 36	-1.33	11.52	-10.17	5.67E-06	0.000519334
262	Myo1f	myosin IF	-1.33	9.89	-6.64	0.000137512	0.003809939
263	Gpr68	G protein-coupled receptor 68	-1.34	8.89	-12.97	8.39E-07	0.000191956
264	Ephx1	epoxide hydrolase 1, microsomal	-1.34	10.81	-8.48	2.28E-05	0.001199946
265	Tacstd2	tumor-associated calcium signal transducer 2	-1.35	8.54	-15.12	2.46E-07	0.000112734
266	Itgad	integrin, alpha D	-1.35	8.45	-6.40	0.000178721	0.004550005
267	Tcf7	transcription factor 7, T cell specific	-1.36	8.57	-6.99	9.46E-05	0.003022162
268	Rgs10	regulator of G-protein signalling 10	-1.36	9.95	-13.03	8.11E-07	0.000189903
269	Sep9	septin 9	-1.37	8.95	-8.86	1.63E-05	0.001013828
270	Cd8b1	CD8 antigen, beta chain 1	-1.37	9.16	-3.66	0.005982465	0.045057589
271	Sema4a	sema domain, immunoglobulin domain (Ig), transmembrane domain (TM) and s	-1.37	10.37	-9.41	1.04E-05	0.000750419
272	Csf3r	colony stimulating factor 3 receptor (granulocyte)	-1.37	8.57	-10.39	4.81E-06	0.00047416
273	F13a1	coagulation factor XIII, A1 subunit	-1.37	8.55	-6.71	0.000126752	0.003600003
274	Tmem51	transmembrane protein 51	-1.38	9.54	-5.82	0.000341886	0.006791276
275	Gpr171	G protein-coupled receptor 171	-1.38	9.82	-17.78	6.65E-08	7.20E-05
276	Glipr2	GLI pathogenesis-related 2	-1.38	10.42	-8.98	1.48E-05	0.000927095
277	Csrp2	cysteine and glycine-rich protein 2	-1.39	8.74	-14.65	3.17E-07	0.000126365
278	Dusp2	dual specificity phosphatase 2	-1.39	9.31	-9.72	8.02E-06	0.000643485
279	Sostdc1	sclerostin domain containing 1	-1.41	8.64	-4.41	0.002053933	0.021870864
280	Ccr6	chemokine (C-C motif) receptor 6	-1.42	9.02	-6.59	0.000144894	0.003946706
281	Igfbp4	insulin-like growth factor binding protein 4	-1.42	8.50	-5.22	0.000711049	0.010897018
282	Sort1	sortilin 1	-1.42	9.14	-9.58	8.99E-06	0.000680709
283	Fpr2	formyl peptide receptor 2	-1.43	11.21	-4.16	0.002929021	0.027754753
284	Fxyd5	FXYD domain-containing ion transport regulator 5	-1.44	11.88	-7.42	6.12E-05	0.002226548
285	Klra4	killer cell lectin-like receptor, subfamily A, member 4	-1.45	8.77	-14.12	4.26E-07	0.000126365
286	Cmc1	COX assembly mitochondrial protein 1	-1.45	10.24	-8.41	2.43E-05	0.001241645
287	Myl4	myosin, light polypeptide 4	-1.45	10.47	-6.37	0.000183946	0.004612847
288	Dennd3	DENN/MADD domain containing 3	-1.46	10.21	-3.97	0.003801274	0.033188335
289	Hsd11b1	hydroxysteroid 11-beta dehydrogenase 1	-1.46	9.49	-14.72	3.06E-07	0.000126365
290	Csf3r	colony stimulating factor 3 receptor (granulocyte)	-1.47	8.43	-17.60	7.20E-08	7.20E-05
291	F2r	coagulation factor II (thrombin) receptor	-1.48	10.49	-7.14	8.10E-05	0.002688196
292	Fxyd5	FXYD domain-containing ion transport regulator 5	-1.48	10.46	-9.09	1.34E-05	0.000886427
293	Klre1	killer cell lectin-like receptor family E member 1	-1.48	8.64	-9.52	9.47E-06	0.000711445
294	Trib2	tribbles homolog 2 (Drosophila)	-1.50	9.82	-8.80	1.72E-05	0.001046831
295	Neurl3	neuralized homolog 3 homolog (Drosophila)	-1.50	8.63	-8.74	1.81E-05	0.001056366
296	Ctnna1	catenin (cadherin associated protein), alpha 1	-1.51	9.75	-15.87	1.67E-07	8.74E-05
297	Bcl11b	B cell leukemia/lymphoma 11B	-1.52	10.28	-5.81	0.000345715	0.006826447
298	Tmem108	transmembrane protein 108	-1.52	8.48	-10.23	5.44E-06	0.000509109
299	Cyp27a1	cytochrome P450, family 27, subfamily a, polypeptide 1	-1.52	9.00	-10.72	3.77E-06	0.00039567
300	Csf1r	colony stimulating factor 1 receptor	-1.52	9.68	-4.30	0.002397836	0.024405413
301	Gpc1	glypican 1	-1.52	9.28	-10.92	3.25E-06	0.000384201
302	Fcer2a	Fc receptor, IgE, low affinity II, alpha polypeptide	-1.55	8.61	-7.02	9.20E-05	0.002950886
303	Gcnt2	glucosaminyl (N-acetyl) transferase 2, I-branching enzyme	-1.55	9.28	-9.91	6.92E-06	0.000592709
304	Zap70	zeta-chain (TCR) associated protein kinase	-1.56	10.13	-12.46	1.15E-06	0.000226433

305	Chst1	carbohydrate (keratan sulfate Gal-6) sulfotransferase 1	-1.56	9.18	-4.94	0.001016143	0.013686077
306	Hvcn1	hydrogen voltage-gated channel 1	-1.56	11.55	-9.26	1.16E-05	0.000820031
307	Cdc42ep3	CDC42 effector protein (Rho GTPase binding) 3	-1.57	9.86	-10.46	4.57E-06	0.000453629
308	Cxcr3	chemokine (C-X-C motif) receptor 3	-1.59	9.84	-8.07	3.31E-05	0.001539334
309	Leprotl1	leptin receptor overlapping transcript-like 1	-1.61	10.19	-12.28	1.30E-06	0.000236954
310	Mmp9	matrix metallopeptidase 9	-1.61	9.03	-4.50	0.001814258	0.020131968
311	Gpr114	G protein-coupled receptor 114	-1.61	9.43	-7.29	6.97E-05	0.002424401
312	Ffar2	free fatty acid receptor 2	-1.61	9.67	-4.83	0.001168508	0.015027774
313	Pacsin1	protein kinase C and casein kinase substrate in neurons 1	-1.62	9.02	-7.53	5.52E-05	0.002097544
314	Lyz1	lysozyme 1	-1.62	14.55	-9.75	7.87E-06	0.000643485
315	Clec7a	C-type lectin domain family 7, member a	-1.62	8.95	-14.82	2.89E-07	0.000126365
316	Clec4d	C-type lectin domain family 4, member d	-1.64	8.80	-7.18	7.80E-05	0.002639989
317	Ngfrap1	nerve growth factor receptor (TNFRSF16) associated protein 1	-1.64	9.86	-8.08	3.27E-05	0.001539334
318	Rab32	RAB32, member RAS oncogene family	-1.64	9.66	-10.97	3.15E-06	0.000382174
319	Mt1	metallothionein 1	-1.65	10.74	-4.34	0.002276487	0.023602341
320	Cyp27a1	cytochrome P450, family 27, subfamily a, polypeptide 1	-1.65	8.74	-9.01	1.44E-05	0.00092333
321	Sgk1	serum/glucocorticoid regulated kinase 1	-1.65	10.28	-7.75	4.46E-05	0.001845096
322	Lgmn	legumain	-1.65	10.49	-7.38	6.39E-05	0.002306549
323	Itk	IL2 inducible T cell kinase	-1.66	9.64	-7.55	5.41E-05	0.002069558
324	Tmem66	transmembrane protein 66	-1.66	11.10	-6.92	0.000101909	0.003130533
325	Sort1	sortilin 1	-1.66	9.30	-10.09	6.06E-06	0.000540025
326	Tmem66	transmembrane protein 66	-1.67	11.01	-4.80	0.001212497	0.015397073
327	Arap3	ArfGAP with RhoGAP domain, ankyrin repeat and PH domain 3	-1.67	9.00	-8.54	2.17E-05	0.001177646
328	Ramp1	receptor (calcitonin) activity modifying protein 1	-1.69	8.75	-7.93	3.77E-05	0.001662267
329	St6galnac2	ST6 (alpha-N-acetyl-neuraminyl-2,3-beta-galactosyl-1,3)-N-acetylgalactosamini	-1.69	9.92	-13.38	6.54E-07	0.000164898
330	Pglyrp1	peptidoglycan recognition protein 1	-1.69	10.42	-4.91	0.001058114	0.014063476
331	Serpinb1a	serine (or cysteine) peptidase inhibitor, clade B, member 1a	-1.70	9.57	-9.08	1.35E-05	0.000886427
332	Ifitm3	interferon induced transmembrane protein 3	-1.72	11.17	-6.49	0.000160495	0.004214998
333	Hp	haptoglobin	-1.72	9.14	-4.28	0.002457471	0.024849662
334	Lmo2	LIM domain only 2	-1.72	11.85	-14.32	3.80E-07	0.000126365
335	Cd3e	CD3 antigen, epsilon polypeptide	-1.74	10.70	-10.78	3.61E-06	0.00039567
336	Tgfbi	transforming growth factor, beta induced	-1.74	9.71	-11.93	1.62E-06	0.000270244
337	Xlr4a	X-linked lymphocyte-regulated 4A	-1.74	11.46	-12.47	1.15E-06	0.000226433
338	Dok2	docking protein 2	-1.79	9.77	-7.16	7.95E-05	0.002664622
339	Zfp608	zinc finger protein 608	-1.79	8.70	-9.44	1.01E-05	0.00074318
340	Cd6	CD6 antigen	-1.80	9.99	-13.13	7.62E-07	0.00018156
341	NA	NA	-1.80	9.99	-8.21	2.90E-05	0.001422116
342	Tnfrsf4	tumor necrosis factor receptor superfamily, member 4	-1.81	10.02	-7.35	6.59E-05	0.002347302
343	Zyx	zyxin	-1.81	12.54	-12.12	1.44E-06	0.000248712
344	Hsd11b1	hydroxysteroid 11-beta dehydrogenase 1	-1.82	9.90	-18.01	5.98E-08	7.20E-05
345	Dusp2	dual specificity phosphatase 2	-1.82	10.38	-11.63	2.00E-06	0.000307633
346	Dgka	diacylglycerol kinase, alpha	-1.82	10.89	-7.18	7.82E-05	0.002642851
347	Lat	linker for activation of T cells	-1.83	10.61	-16.23	1.39E-07	8.74E-05
348	Tiam1	T cell lymphoma invasion and metastasis 1	-1.83	9.29	-10.33	5.03E-06	0.000491624

349	Trf	transferrin	-1.85	9.00	-6.84	0.000111008	0.003276496
350	NA	NA	-1.87	8.91	-8.06	3.35E-05	0.001544265
351	Gadd45g	growth arrest and DNA-damage-inducible 45 gamma	-1.90	10.08	-9.22	1.21E-05	0.000832985
352	Bcl6	B cell leukemia/lymphoma 6	-1.91	10.44	-8.08	3.29E-05	0.001539334
353	Dgka	diacylglycerol kinase, alpha	-1.91	11.54	-6.59	0.000144058	0.003943894
354	Il18r1	interleukin 18 receptor 1	-1.93	9.89	-17.37	8.04E-08	7.20E-05
355	C1qc	complement component 1, q subcomponent, C chain	-1.93	9.22	-11.44	2.27E-06	0.000333942
356	Fcgr3	Fc receptor, IgG, low affinity III	-1.93	9.07	-5.93	0.000302045	0.006253232
357	Cd6	CD6 antigen	-1.94	10.43	-11.40	2.32E-06	0.000338092
358	Hsd11b1	hydroxysteroid 11-beta dehydrogenase 1	-1.96	10.48	-23.34	7.23E-09	1.90E-05
359	Il7r	interleukin 7 receptor	-1.96	10.42	-9.99	6.53E-06	0.000571
360	Cd3g	CD3 antigen, gamma polypeptide	-1.98	11.13	-11.02	3.05E-06	0.000376611
361	Pxdc1	PX domain containing 1	-1.98	9.32	-9.14	1.29E-05	0.000864652
362	Tmem176b	transmembrane protein 176B	-2.00	9.68	-12.43	1.17E-06	0.000226433
363	Cd8b1	CD8 antigen, beta chain 1	-2.02	12.18	-5.97	0.000289485	0.006114237
364	Igfbp4	insulin-like growth factor binding protein 4	-2.04	8.88	-7.02	9.21E-05	0.002950886
365	Cd6	CD6 antigen	-2.05	10.96	-9.43	1.02E-05	0.00074318
366	Emb	embigin	-2.06	9.96	-15.10	2.49E-07	0.000112734
367	Ear4	eosinophil-associated, ribonuclease A family, member 4	-2.07	9.25	-8.97	1.48E-05	0.000927095
368	Fcer2a	Fc receptor, IgE, low affinity II, alpha polypeptide	-2.07	9.04	-9.09	1.35E-05	0.000886427
369	Cd27	CD27 antigen	-2.08	11.11	-17.27	8.41E-08	7.20E-05
370	Klk8	kallikrein related-peptidase 8	-2.09	9.80	-8.60	2.05E-05	0.001143744
371	Ifitm6	interferon induced transmembrane protein 6	-2.10	9.77	-5.07	0.000859724	0.012443619
372	C1qb	complement component 1, q subcomponent, beta polypeptide	-2.10	9.58	-11.12	2.82E-06	0.000365287
373	Lyz2	lysozyme 2	-2.12	12.10	-15.93	1.62E-07	8.74E-05
374	Ly6c1	lymphocyte antigen 6 complex, locus C1	-2.14	10.49	-6.17	0.000228945	0.005376928
375	Lgals3	lectin, galactose binding, soluble 3	-2.15	11.95	-11.17	2.73E-06	0.000364587
376	Stat4	signal transducer and activator of transcription 4	-2.17	10.16	-10.82	3.52E-06	0.00039538
377	B3gnt8	UDP-GlcNAc:betaGal beta-1,3-N-acetylglucosaminyltransferase 8	-2.17	10.18	-14.13	4.23E-07	0.000126365
378	Cd3d	CD3 antigen, delta polypeptide	-2.18	10.96	-13.89	4.87E-07	0.000130244
379	Sirpb1a	signal-regulatory protein beta 1A	-2.19	9.88	-16.08	1.50E-07	8.74E-05
380	Ccr6	chemokine (C-C motif) receptor 6	-2.19	10.34	-5.75	0.000374546	0.007176066
381	Il4i1	interleukin 4 induced 1	-2.22	11.72	-7.69	4.75E-05	0.00190209
382	Thy1	thymus cell antigen 1, theta	-2.24	10.76	-10.21	5.49E-06	0.000510469
383	Ifitm2	interferon induced transmembrane protein 2	-2.25	10.80	-10.10	6.01E-06	0.000539159
384	Ccl9	chemokine (C-C motif) ligand 9	-2.27	9.39	-13.17	7.42E-07	0.000180048
385	Dpp4	dipeptidylpeptidase 4	-2.28	10.18	-11.69	1.91E-06	0.000303211
386	Ctsw	cathepsin W	-2.28	10.26	-14.53	3.40E-07	0.000126365
387	Klrd1	killer cell lectin-like receptor, subfamily D, member 1	-2.29	9.84	-10.70	3.83E-06	0.00039567
388	Il1b	interleukin 1 beta	-2.31	9.62	-12.15	1.41E-06	0.000246908
389	Axl	AXL receptor tyrosine kinase	-2.33	9.65	-9.10	1.33E-05	0.000885286
390	Lyz2	lysozyme 2	-2.33	13.02	-10.27	5.28E-06	0.0005051
391	Hp	haptoglobin	-2.34	9.83	-5.82	0.000344727	0.006824249
392	S100a9	S100 calcium binding protein A9 (calgranulin B)	-2.35	12.85	-4.01	0.003592089	0.031897675

393	Ear2	eosinophil-associated, ribonuclease A family, member 2	-2.44	9.87	-10.97	3.14E-06	0.000382174
394	Prg2	proteoglycan 2, bone marrow	-2.50	9.43	-26.42	2.61E-09	1.14E-05
395	Vcam1	vascular cell adhesion molecule 1	-2.53	9.44	-10.93	3.24E-06	0.000384201
396	Alox5ap	arachidonate 5-lipoxygenase activating protein	-2.53	10.63	-12.50	1.12E-06	0.000226433
397	Chchd10	coiled-coil-helix-coiled-coil-helix domain containing 10	-2.60	11.67	-23.74	6.28E-09	1.90E-05
398	Chi3l3	chitinase 3-like 3	-2.63	10.46	-3.92	0.004099802	0.034940263
399	Fcna	ficolin A	-2.74	9.51	-14.52	3.40E-07	0.000126365
400	Fcer2a	Fc receptor, IgE, low affinity II, alpha polypeptide	-2.90	9.99	-9.64	8.61E-06	0.000659489
401	Satb1	special AT-rich sequence binding protein 1	-2.92	11.26	-9.26	1.17E-05	0.000820031
402	Slc40a1	solute carrier family 40 (iron-regulated transporter), member 1	-2.98	10.90	-16.03	1.54E-07	8.74E-05
403	Vpreb3	pre-B lymphocyte gene 3	-3.59	10.07	-26.45	2.58E-09	1.14E-05
404	Slpi	secretory leukocyte peptidase inhibitor	-3.67	11.58	-6.89	0.000105045	0.003186622

Column headings:

LogFC: Log2-fold of change between TRAF3-/- B lymphomas and LMC spleens

AveExpr: average expression level for both conditions (LMC and TRAF3-/-)

t: the t-statistic

P. Value: the p value based on the t-statistic

Adj. P. Val.: the p value adjusted for multiple measurements (essentially the false discovery rate)

The mRNA expression profiles of splenocytes from LMC and tumor-bearing B-TRAF3-/- mice (mouse ID: 6983-2, 7041-10, and 7060-8) were analyzed by a microarray analysis. cRNA was hybridized to Illumina Sentrix Mouse Whole Genome 24K Microarray (Illumina). We determined two group comparisons of the normalized data for triplicate samples using paired t tests and false discovery rate. Genes listed include 160 up-regulated and 244 down-regulated genes (fold of change: >2).
