# Supplementary material for: Mutated in colorectal cancer (MCC) is a novel oncogene in B lymphocytes
Source: J Hematol Oncol. 2014 Sep 9;7:56. doi: 10.1186/s13045-014-0056-6 (PMC4172902; doi:10.1186/s13045-014-0056-6)
Supplement: Additional file 2: Table S2. — The MCC-interactome in human MM cells identified by affinity purification followed by LC-MS/MS. [file 13045_2014_56_MOESM2_ESM.pdf]

Supplementary Table 2. The MCC-interactome in human MM cells identified by affinity purification followed by LC-MS/MS

#	Accession	Description	SwissProt ID	Average spectral count difference (hMCC-SBP-6xHis - FLAG-hMCC) Mitochondria	Whole lysates	Previously known MCC-interactor
NA	P23508	Colorectal mutant cancer protein	CRCM_HUMAN	415	1223	Not applicable
1	P23508-2	Isoform 2 of Colorectal mutant cancer protein	CRCM_HUMAN	395.5	1172.5	No
2	P09874	Poly [ADP-ribose] polymerase 1	PARP1_HUMAN	72	43.5	No
3	O15020-2	Isoform 2 of Spectrin beta chain, non-erythrocytic 2	SPTN2_HUMAN	34	3	No
4	P40939	Trifunctional enzyme subunit alpha, mitochondrial	ECHA_HUMAN	31	24	No
5	P05141	ADP/ATP translocase 2	ADT2_HUMAN	24	33.5	No
6	P12236	ADP/ATP translocase 3	ADT3_HUMAN	20.5	27.5	No
7	P06576	ATP synthase subunit beta, mitochondrial	ATPB_HUMAN	18.5	19.5	No
8	Q99623	Prohibitin-2	PHB2_HUMAN	17	23.5	Yes (Ewing, 2007)
9	Q13576	Ras GTPase-activating-like protein IQGAP2	IQGA2_HUMAN	16	50	No
10	P12235	ADP/ATP translocase 1	ADT1_HUMAN	15.5	24	No
11	Q9Y277	Voltage-dependent anion-selective channel protein 3	VDAC3_HUMAN	15.5	2.5	No
12	Q02880-2	Isoform Beta-1 of DNA topoisomerase 2-beta	TOP2B_HUMAN	14.5	12.5	No
13	O60264	SWI/SNF-related matrix-associated actin-dependent regulator (	SMCA5_HUMAN	13.5	2	No
14	P35232	Prohibitin	PHB_HUMAN	13	3.5	No
15	O00571	ATP-dependent RNA helicase DDX3X	DDX3X_HUMAN	12	17.5	No
16	P13674	Prolyl 4-hydroxylase subunit alpha-1	P4HA1_HUMAN	12	9	No
17	Q9Y2X3	Nucleolar protein 58	NOP58_HUMAN	11.5	10.5	No
18	P13674-2	Isoform 2 of Prolyl 4-hydroxylase subunit alpha-1	P4HA1_HUMAN	11	8.5	No
19	P16403	Histone H1.2	H12_HUMAN	9.5	8.5	Yes (Sigglekow, 2012)
20	P13796	Plastin-2	PLSL_HUMAN	9.5	7.5	No
21	P42166	Lamina-associated polypeptide 2, isoform alpha	LAP2A_HUMAN	9	19.5	No
22	P46940	Ras GTPase-activating-like protein IQGAP1	IQGA1_HUMAN	8	31	No
23	P47756-2	Isoform 2 of F-actin-capping protein subunit beta	CAPZB_HUMAN	7.5	6.5	Yes (Ewing, 2007)
24	P06493	Cyclin-dependent kinase 1	CDK1_HUMAN	7	8	No
25	P12956	X-ray repair cross-complementing protein 6	XRCC6_HUMAN	7	6.5	No
26	P35251-2	Isoform 2 of Replication factor C subunit 1	RFC1_HUMAN	7	3.5	No
27	Q14974	Importin subunit beta-1	IMB1_HUMAN	6.5	18	No
28	P78371	T-complex protein 1 subunit beta	TCPB_HUMAN	6.5	12	No
29	Q9Y5B9	FACT complex subunit SPT16	SP16H_HUMAN	6.5	6	Yes (Ewing, 2007)
30	Q86UE4	Protein LYRIC	LYRIC_HUMAN	6.5	3.5	No
31	Q9BVP2-2	Isoform 2 of Guanine nucleotide-binding protein-like 3	GNL3_HUMAN	6.5	1	No
32	Q5T4S7-3	Isoform 3 of E3 ubiquitin-protein ligase UBR4	UBR4_HUMAN	6	69	No
33	Q96ER9	Coiled-coil domain-containing protein 51	CCD51_HUMAN	6	17	No
34	P31689	DnaJ homolog subfamily A member 1	DNJA1_HUMAN	6	14.5	No
35	P63092	Guanine nucleotide-binding protein G(s) subunit alpha isoforms	GNAS2_HUMAN	6	5.5	No
36	P53621	Coatomer subunit alpha	COPA_HUMAN	6	5	No
37	P22732	Solute carrier family 2, facilitated glucose transporter member 5	GTR5_HUMAN	6	4	No

38	P63092-2	Isoform Gnas-2 of Guanine nucleotide-binding protein G(s) sub	GNAS2_HUMAN	5.5	5.5	No
39	P63092-3	Isoform 3 of Guanine nucleotide-binding protein G(s) subunit al	GNAS2_HUMAN	5.5	5	No
40	Q14839	Chromodomain-helicase-DNA-binding protein 4	CHD4_HUMAN	5.5	4.5	No
41	Q8NI36	WD repeat-containing protein 36	WDR36_HUMAN	5.5	4	No
42	P17858	6-phosphofructokinase, liver type	K6PL_HUMAN	5	6.5	No
43	P30041	Peroxiredoxin-6	PRDX6_HUMAN	5	6	No
44	Q9P035	Very-long-chain (3R)-3-hydroxyacyl-[acyl-carrier protein] dehydı	HACD3_HUMAN	5	6	No
45	P18754	Regulator of chromosome condensation	RCC1_HUMAN	5	4.5	No
46	Q9BSD7	Cancer-related nucleoside-triphosphatase	NTPCR_HUMAN	5	4	No
47	P62873	Guanine nucleotide-binding protein G(I)/G(S)/G(T) subunit beta	GBB1_HUMAN	5	3.5	Yes (Ewing, 2007)
48	O94874	E3 UFM1-protein ligase 1	UFL1_HUMAN	5	3	No
49	O95299	NADH dehydrogenase [ubiquinone] 1 alpha subcomplex subun	NDUAA_HUMAN	5	3	No
50	Q9UQE7	Structural maintenance of chromosomes protein 3	SMC3_HUMAN	4.5	24	Yes (Ewing, 2007)
51	P62136	Serine/threonine-protein phosphatase PP1-alpha catalytic subu	PP1A_HUMAN	4.5	8.5	No
52	Q14157	Ubiquitin-associated protein 2-like	UBP2L_HUMAN	4.5	7	No
53	O75534-2	Isoform Short of Cold shock domain-containing protein E1	CSDE1_HUMAN	4.5	5	Yes (Ewing, 2007)
54	Q00013	55 kDa erythrocyte membrane protein	EM55_HUMAN	4.5	2.5	No
55	P17480-2	Isoform UBF2 of Nucleolar transcription factor 1	UBF1_HUMAN	4.5	2.5	No
56	Q01813	6-phosphofructokinase type C	K6PP_HUMAN	4.5	2	Yes (Ewing, 2007)
57	Q9UIG0-2	Isoform 2 of Tyrosine-protein kinase BAZ1B	BAZ1B_HUMAN	4.5	2	No
58	P33991	DNA replication licensing factor MCM4	MCM4_HUMAN	4	27	Yes (Ewing, 2007)
59	Q92973-2	Isoform 2 of Transportin-1	TNPO1_HUMAN	4	15.5	No
60	Q08945	FACT complex subunit SSRP1	SSRP1_HUMAN	4	9.5	Yes (Ewing, 2007)
61	Q13242	Serine/arginine-rich splicing factor 9	SRSF9_HUMAN	4	5	No
62	P23528	Cofilin-1	COF1_HUMAN	4	4	No
63	Q92922	SWI/SNF complex subunit SMARCC1	SMRC1_HUMAN	4	3.5	No
64	Q14699	Raftlin	RFTN1_HUMAN	4	3	No
65	O94826	Mitochondrial import receptor subunit TOM70	TOM70_HUMAN	4	2.5	No
66	P51531-2	Isoform Short of Probable global transcription activator SNF2L2	SMCA2_HUMAN	4	2.5	No
67	Q9UQ80	Proliferation-associated protein 2G4	PA2G4_HUMAN	4	2	No
68	O43396	Thioredoxin-like protein 1	TXNL1_HUMAN	3.5	15.5	No
69	O60884	DnaJ homolog subfamily A member 2	DNJA2_HUMAN	3.5	10	No
70	P36873	Serine/threonine-protein phosphatase PP1-gamma catalytic sul	PP1G_HUMAN	3.5	8.5	No
71	P53985	Monocarboxylate transporter 1	MOT1_HUMAN	3.5	8.5	No
72	P40938	Replication factor C subunit 3	RFC3_HUMAN	3.5	4.5	No
73	Q53HL2	Borealin	BOREA_HUMAN	3.5	4	No
74	P21912	Succinate dehydrogenase [ubiquinone] iron-sulfur subunit, mito	DHSB_HUMAN	3.5	3.5	Yes (Ewing, 2007)
75	Q86WU2-2	Isoform 2 of Probable D-lactate dehydrogenase, mitochondrial	LDHD_HUMAN	3.5	3.5	No
76	Q96JB5	CDK5 regulatory subunit-associated protein 3	CK5P3_HUMAN	3.5	3	No
77	O95563	Mitochondrial pyruvate carrier 2	MPC2_HUMAN	3.5	2.5	No
78	Q96QK1	Vacuolar protein sorting-associated protein 35	VPS35_HUMAN	3.5	2.5	Yes (Ewing, 2007)
79	Q9BZQ8	Protein Niban	NIBAN_HUMAN	3.5	1.5	No
80	O14773-2	Isoform 2 of Tripeptidyl-peptidase 1	TPP1_HUMAN	3.5	1.5	No
81	Q92945	Far upstream element-binding protein 2	FUBP2_HUMAN	3	7.5	No

82	Q9P0L0	Vesicle-associated membrane protein-associated protein A	VAPA_HUMAN	3	6	No
83	Q15181	Inorganic pyrophosphatase	IPYR_HUMAN	3	5.5	No
84	Q12788	Transducin beta-like protein 3	TBL3_HUMAN	3	5	No
85	O95573	Long-chain-fatty-acid--CoA ligase 3	ACSL3_HUMAN	3	4.5	No
86	Q02978	Mitochondrial 2-oxoglutarate/malate carrier protein	M2OM_HUMAN	3	3	No
87	Q9UKM7	Endoplasmic reticulum mannosyl-oligosaccharide 1,2-alpha-ma	MA1B1_HUMAN	3	3	No
88	Q7Z3B4	Nucleoporin p54	NUP54_HUMAN	3	2	No
89	P42224	Signal transducer and activator of transcription 1-alpha/beta	STAT1_HUMAN	3	2	No
90	Q99733	Nucleosome assembly protein 1-like 4	NP1L4_HUMAN	3	2	No
91	P35998	26S protease regulatory subunit 7	PRS7_HUMAN	2.5	10	No
92	P12004	Proliferating cell nuclear antigen	PCNA_HUMAN	2.5	8.5	Yes (Ewing, 2007)
93	O00299	Chloride intracellular channel protein 1	CLIC1_HUMAN	2.5	8.5	Yes (Ewing, 2007)
94	O75083	WD repeat-containing protein 1	WDR1_HUMAN	2.5	5.5	No
95	Q9Y3F4	Serine-threonine kinase receptor-associated protein	STRAP_HUMAN	2.5	5	No
96	P43358	Melanoma-associated antigen 4	MAGA4_HUMAN	2.5	5	No
97	Q08752	Peptidyl-prolyl cis-trans isomerase D	PPID_HUMAN	2.5	5	No
98	O43175	D-3-phosphoglycerate dehydrogenase	SERA_HUMAN	2.5	4.5	No
99	Q9GZS3	WD repeat-containing protein 61	WDR61_HUMAN	2.5	4	No
100	P53701	Cytochrome c-type heme lyase	CCHL_HUMAN	2.5	4	No
101	Q14258	E3 ubiquitin/ISG15 ligase TRIM25	TRI25_HUMAN	2.5	3.5	No
102	P35249	Replication factor C subunit 4	RFC4_HUMAN	2.5	3	No
103	P51159	Ras-related protein Rab-27A	RB27A_HUMAN	2.5	3	No
104	P07741	Adenine phosphoribosyltransferase]	APT_HUMAN	2.5	2.5	Yes (Ewing, 2007)
105	Q96HE7	ERO1-like protein alpha	ERO1A_HUMAN	2.5	2.5	No
106	Q8WYP5	Protein ELYS	ELYS_HUMAN	2.5	2.5	No
107	Q9H0U3	Magnesium transporter protein 1	MAGT1_HUMAN	2.5	1.5	No
108	O15400-2	Isoform 2 of Syntaxin-7	STX7_HUMAN	2.5	1	No
109	Q9Y5X1	Sorting nexin-9	SNX9_HUMAN	2.5	1	No
110	Q969V3-2	Isoform 2 of Nicalin	NCLN_HUMAN	2.5	1	No
111	Q9BQG0	Myb-binding protein 1A	MBB1A_HUMAN	2.5	1	No
112	P38117	Electron transfer flavoprotein subunit beta	ETFB_HUMAN	2	14	No
113	Q9BYG3	MKI67 FHA domain-interacting nucleolar phosphoprotein	MK67I_HUMAN	2	9	No
114	Q9NX58	Cell growth-regulating nucleolar protein	LYAR_HUMAN	2	9	No
115	Q9BPW8	Protein NipSnap homolog 1	NIPS1_HUMAN	2	8	No
116	Q8IYB3	Serine/arginine repetitive matrix protein 1	SRRM1_HUMAN	2	8	No
117	P69849	Nodal modulator 3	NOMO3_HUMAN	2	7.5	No
118	Q14203-5	Isoform 5 of Dynactin subunit 1	DCTN1_HUMAN	2	6.5	No
119	Q6DD88	Atlastin-3	ATLA3_HUMAN	2	6.5	No
120	P53004	Biliverdin reductase A	BIEA_HUMAN	2	6.5	No
121	O60763	General vesicular transport factor p115	USO1_HUMAN	2	5	No
122	P13010	X-ray repair cross-complementing protein 5	XRCC5_HUMAN	2	5	No
123	Q16836	Hydroxyacyl-coenzyme A dehydrogenase, mitochondrial	HCDH_HUMAN	2	4.5	Yes (Ewing, 2007)
124	Q09028-3	Isoform 3 of Histone-binding protein RBBP4	RBBP4_HUMAN	2	4	No
125	Q15126	Phosphomevalonate kinase	PMVK_HUMAN	2	3.5	No

126	P18669	Phosphoglycerate mutase 1	PGAM1_HUMAN	2	3.5	No
127	P23258	Tubulin gamma-1 chain	TBG1_HUMAN	2	3.5	No
128	Q9HC21	Mitochondrial thiamine pyrophosphate carrier	TPC_HUMAN	2	3.5	No
129	P61160	Actin-related protein 2	ARP2_HUMAN	2	3	No
130	Q12769	Nuclear pore complex protein Nup160	NU160_HUMAN	2	2.5	No
131	Q96HY6	DDRGK domain-containing protein 1	DDRGK_HUMAN	2	2	No
132	P48960-2	Isoform 2 of CD97 antigen	CD97_HUMAN	2	2	No
133	Q13610	Periodic tryptophan protein 1 homolog	PWP1_HUMAN	2	1.5	No
134	Q9H7Z7	Prostaglandin E synthase 2	PGES2_HUMAN	2	1.5	No
135	Q14008-2	Isoform 2 of Cytoskeleton-associated protein 5	CKAP5_HUMAN	2	1	No
136	Q14C86-4	Isoform 4 of GTPase-activating protein and VPS9 domain-conta	GAPD1_HUMAN	1.5	29.5	No
137	P62937	Peptidyl-prolyl cis-trans isomerase A	PPIA_HUMAN	1.5	14.5	No
138	Q99798	Aconitate hydratase, mitochondrial	ACON_HUMAN	1.5	8.5	No
139	O43291	Kunitz-type protease inhibitor 2	SPIT2_HUMAN	1.5	6	No
140	Q02790	Peptidyl-prolyl cis-trans isomerase FKBP4	FKBP4_HUMAN	1.5	5	No
141	Q15397	Pumilio domain-containing protein KIAA0020	K0020_HUMAN	1.5	5	No
142	Q96SB4	SRSF protein kinase 1	SRPK1_HUMAN	1.5	5	No
143	P46977	Dolichyl-diphosphooligosaccharide--protein glycosyltransferase	STT3A_HUMAN	1.5	4.5	No
144	P07686	Beta-hexosaminidase subunit beta	HEXB_HUMAN	1.5	4	No
145	Q6P1M0	Long-chain fatty acid transport protein 4	S27A4_HUMAN	1.5	3.5	No
146	P11532-3	Isoform 2 of Dystrophin	DMD_HUMAN	1.5	2.5	No
147	Q9NQS7-2	Isoform 2 of Inner centromere protein	INCE_HUMAN	1.5	2.5	No
148	O96008	Mitochondrial import receptor subunit TOM40 homolog	TOM40_HUMAN	1.5	2	No
149	O60488-2	Isoform Short of Long-chain-fatty-acid--CoA ligase 4	ACSL4_HUMAN	1.5	2	No
150	Q9UL25	Ras-related protein Rab-21	RAB21_HUMAN	1.5	2	Yes (Ewing, 2007)
151	Q9Y2W1	Thyroid hormone receptor-associated protein 3	TR150_HUMAN	1	25	No
152	Q9Y3T9	Nucleolar complex protein 2 homolog	NOC2L_HUMAN	1	7	No
153	P07195	L-lactate dehydrogenase B chain	LDHB_HUMAN	1	6.5	No
154	Q13243	Serine/arginine-rich splicing factor 5	SRSF5_HUMAN	1	6.5	No
155	P22061	Protein-L-isoaspartate(D-aspartate) O-methyltransferase	PIMT_HUMAN	1	5.5	Yes (Ewing, 2007)
156	Q5JTV8	Torsin-1A-interacting protein 1 OS=Homo sapiens GN=TOR1A	TOIP1_HUMAN	1	5.5	No
157	O75494-5	Isoform 5 of Serine/arginine-rich splicing factor 10	SRS10_HUMAN	1	5.5	No
158	P35914	Hydroxymethylglutaryl-CoA lyase, mitochondrial	HMGCL_HUMAN	1	5	No
159	Q9H9B4	Sideroflexin-1	SFXN1_HUMAN	1	4.5	Yes (Ewing, 2007)
160	O00487	26S proteasome non-ATPase regulatory subunit 14	PSDE_HUMAN	1	4.5	Yes (Ewing, 2007)
161	O43684-2	Isoform 2 of Mitotic checkpoint protein BUB3	BUB3_HUMAN	1	4	No
162	Q9UNQ2	Probable dimethyladenosine transferase	DIM1_HUMAN	1	4	No
163	Q8ND30	Liprin-beta-2	LIPB2_HUMAN	1	4	No
164	Q96F07-2	Isoform 2 of Cytoplasmic FMR1-interacting protein 2	CYFP2_HUMAN	1	4	No
165	Q9NTK5	Obg-like ATPase 1	OLA1_HUMAN	1	4	No
166	P62879	Guanine nucleotide-binding protein G(I)/G(S)/G(T) subunit beta	GBB2_HUMAN	1	3.5	No
167	Q9UKG1	DCC-interacting protein 13-alpha	DP13A_HUMAN	1	3.5	No
168	Q9Y5K5-2	Isoform 2 of Ubiquitin carboxyl-terminal hydrolase isozyme L5	UCHL5_HUMAN	1	3	No
169	P61964	WD repeat-containing protein 5	WDR5_HUMAN	1	3	Yes (Ewing, 2007)

170	Q8NE71-2	Isoform 2 of ATP-binding cassette sub-family F member 1	ABCF1_HUMAN	1	3	No
171	Q9BQ75-2	Isoform 2 of Protein CMSS1	CMS1_HUMAN	1	2.5	No
172	Q14764	Major vault protein	MVP_HUMAN	1	2.5	No
173	Q96N66-2	Isoform 2 of Lysophospholipid acyltransferase 7	MBOA7_HUMAN	1	2.5	No
174	O75477	Erlin-1	ERLN1_HUMAN	1	2.5	No
175	Q9Y679-2	Isoform Short of Ancient ubiquitous protein 1	AUP1_HUMAN	1	2	No
176	Q15149-4	Isoform 4 of Plectin	PLEC_HUMAN	61.5	0	No
177	P33527-4	Isoform 4 of Multidrug resistance-associated protein 1	MRP1_HUMAN	9.5	0	No
178	Q8N884	Cyclic GMP-AMP synthase	CGAS_HUMAN	9	0.5	No
179	P43121	Cell surface glycoprotein MUC18	MUC18_HUMAN	6.5	0	No
180	P31946-2	Isoform Short of 14-3-3 protein beta/alpha	1433B_HUMAN	6	0	No
181	O00161-2	Isoform SNAP-23b of Synaptosomal-associated protein 23	SNP23_HUMAN	5.5	0.5	No
182	Q9BUR5	Apolipoprotein O	APOO_HUMAN	5.5	0.5	No
183	P46459	Vesicle-fusing ATPase	NSF_HUMAN	5	0	No
184	Q04917	14-3-3 protein eta	1433F_HUMAN	4.5	0.5	No
185	Q7Z403	Transmembrane channel-like protein 6	TMC6_HUMAN	4.5	0	No
186	Q92508	Piezo-type mechanosensitive ion channel component 1	PIEZ1_HUMAN	4.5	0	No
187	Q8NC56	LEM domain-containing protein 2	LEMD2_HUMAN	4	0.5	No
188	P31947-2	Isoform 2 of 14-3-3 protein sigma	1433S_HUMAN	4	0	No
189	Q9C0B5-2	Isoform 2 of Palmitoyltransferase ZDHHC5	ZDHC5_HUMAN	3.5	0	No
190	Q8IV63-3	Isoform 3 of Inactive serine/threonine-protein kinase VRK3	VRK3_HUMAN	3.5	0	No
191	Q8NBJ5	Procollagen galactosyltransferase 1	GT251_HUMAN	3.5	0	No
192	O96000	NADH dehydrogenase [ubiquinone] 1 beta subcomplex subunit	NDUBA_HUMAN	3	0.5	No
193	Q9Y5Y6	Suppressor of tumorigenicity 14 protein	ST14_HUMAN	3	0	No
194	Q96S97	Myeloid-associated differentiation marker	MYADM_HUMAN	3	0	No
195	Q92485	Acid sphingomyelinase-like phosphodiesterase 3b	ASM3B_HUMAN	3	0	No
196	Q13185	Chromobox protein homolog 3	CBX3_HUMAN	3	0	No
197	Q9H078-2	Isoform 2 of Caseinolytic peptidase B protein homolog	CLPB_HUMAN	2.5	0.5	No
198	Q8NBI5	Solute carrier family 43 member 3	S43A3_HUMAN	2.5	0	No
199	O94776	Metastasis-associated protein MTA2	MTA2_HUMAN	2.5	0	No
200	Q13425-2	Isoform 2 of Beta-2-syntrophin	SNTB2_HUMAN	2.5	0	No
201	Q13951-2	Isoform 2 of Core-binding factor subunit beta	PEBB_HUMAN	2.5	0	No
202	Q99595	Mitochondrial import inner membrane translocase subunit Tim1	TI17A_HUMAN	2.5	0	No
203	Q8TB52	F-box only protein 30	FBX30_HUMAN	2	0	No
204	Q6PJF5-2	Isoform 2 of Inactive rhomboid protein 2	RHDF2_HUMAN	2	0	No
205	Q9H4I3	TraB domain-containing protein	TRABD_HUMAN	2	0	No
206	Q15599-2	Isoform 2 of Na(+)/H(+) exchange regulatory cofactor NHE-RF2	NHRF2_HUMAN	2	0	No
207	Q9H061	Transmembrane protein 126A	T126A_HUMAN	2	0	No
208	P12270	Nucleoprotein TPR	TPR_HUMAN	0	22	No
209	Q9H307	Pinin	PININ_HUMAN	0	18.5	No
210	Q9NYF8-2	Isoform 2 of Bcl-2-associated transcription factor 1	BCLF1_HUMAN	0	18	No
211	P49674	Casein kinase I isoform epsilon	KC1E_HUMAN	0	15.5	Yes (Ewing, 2007)
212	Q9UQ35	Serine/arginine repetitive matrix protein 2	SRRM2_HUMAN	0	15.5	No
213	Q14980-2	Isoform 2 of Nuclear mitotic apparatus protein 1	NUMA1_HUMAN	0.5	14.5	No

214	Q86V48-2	Isoform 2 of Leucine zipper protein 1	LUZP1_HUMAN	0	14.5	No
215	P48730-2	Isoform 2 of Casein kinase I isoform delta	KC1D_HUMAN	0	14.5	Yes (Ewing, 2007)
216	Q9UKV3	Apoptotic chromatin condensation inducer in the nucleus	ACINU_HUMAN	0.5	14	No
217	Q14978-3	Isoform 3 of Nucleolar and coiled-body phosphoprotein 1	NOLC1_HUMAN	0	13	No
218	Q14978	Nucleolar and coiled-body phosphoprotein 1	NOLC1_HUMAN	0	13	No
219	P02788-2	Isoform DeltaLf of Lactotransferrin	TRFL_HUMAN	0	11.5	No
220	P27816-6	Isoform 6 of Microtubule-associated protein 4	MAP4_HUMAN	0.5	10	No
221	Q8N163	DBIRD complex subunit KIAA1967	K1967_HUMAN	0	10	No
222	O75828	Carbonyl reductase [NADPH] 3	CBR3_HUMAN	0	9.5	No
223	P05109	Protein S100-A8	S10A8_HUMAN	0.5	9	No
224	Q9H2U2	Inorganic pyrophosphatase 2, mitochondrial	IPYR2_HUMAN	0.5	8.5	No
225	O75691	Small subunit processome component 20 homolog	UTP20_HUMAN	0	8.5	No
226	P62191	26S protease regulatory subunit 4	PRS4_HUMAN	0	8	No
227	Q96R06	Sperm-associated antigen 5	SPAG5_HUMAN	0	8	No
228	Q15154	Pericentriolar material 1 protein	PCM1_HUMAN	0	8	No
229	Q9BZQ6	ER degradation-enhancing alpha-mannosidase-like protein 3	EDEM3_HUMAN	0.5	7.5	No
230	Q13595	Transformer-2 protein homolog alpha	TRA2A_HUMAN	0.5	7	No
231	Q9NZ01	Very-long-chain enoyl-CoA reductase	TECR_HUMAN	0	7	No
232	Q9NY61	Protein AATF	AATF_HUMAN	0	7	No
233	P46109	Crk-like protein	CRKL_HUMAN	0	6.5	No
234	O75663	TIP41-like protein	TIPRL_HUMAN	0	6.5	No
235	Q06323	Proteasome activator complex subunit 1	PSME1_HUMAN	0	6.5	No
236	P29590-14	Isoform PML-14 of Protein PML	PML_HUMAN	0	6.5	No
237	Q9P0J7	E3 ubiquitin-protein ligase KCMF1	KCMF1_HUMAN	0	6.5	No
238	Q8IZT6-2	Isoform 2 of Abnormal spindle-like microcephaly-associated pro	ASPM_HUMAN	0	6.5	No
239	P49821-2	Isoform 2 of NADH dehydrogenase [ubiquinone] flavoprotein 1,	NDUV1_HUMAN	0.5	6	No
240	P45974-2	Isoform Short of Ubiquitin carboxyl-terminal hydrolase 5	UBP5_HUMAN	0.5	6	No
241	Q5SRE5-2	Isoform 2 of Nucleoporin NUP188 homolog	NU188_HUMAN	0.5	6	No
242	P51114	Fragile X mental retardation syndrome-related protein 1	FXR1_HUMAN	0	6	No
243	Q9H9E3	Conserved oligomeric Golgi complex subunit 4	COG4_HUMAN	0	6	No
244	P29353-2	Isoform p52Shc of SHC-transforming protein 1	SHC1_HUMAN	-0.5	6	No
245	Q92597	Protein NDRG1	NDRG1_HUMAN	-0.5	6	No
246	Q9BRK5	45 kDa calcium-binding protein	CAB45_HUMAN	0.5	5.5	No
247	P50897	Palmitoyl-protein thioesterase 1	PPT1_HUMAN	0.5	5.5	No
248	Q6NVY1	3-hydroxyisobutyryl-CoA hydrolase, mitochondrial	HIBCH_HUMAN	0.5	5.5	No
249	O00170	AH receptor-interacting protein	AIP_HUMAN	0	5.5	No
250	Q9NVN8	Guanine nucleotide-binding protein-like 3-like protein	GNL3L_HUMAN	0	5.5	No
251	Q00534	Cyclin-dependent kinase 6	CDK6_HUMAN	0	5.5	No
252	Q96FW1	Ubiquitin thioesterase OTUB1	OTUB1_HUMAN	0	5	Yes (Ewing, 2007)
253	Q7LBC6	Lysine-specific demethylase 3B	KDM3B_HUMAN	0	5	No
254	Q5VT06	Centrosome-associated protein 350	CE350_HUMAN	0	5	No
255	Q8TC07-2	Isoform 2 of TBC1 domain family member 15	TBC15_HUMAN	0	5	Yes (Ewing, 2007)
256	P54819-5	Isoform 5 of Adenylate kinase 2, mitochondrial	KAD2_HUMAN	0.5	4.5	Yes (Ewing, 2007)
257	Q6FI81-3	Isoform 3 of Anamorsin	CPIN1_HUMAN	0.5	4.5	No

258	Q8WUM0	Nuclear pore complex protein Nup133	NU133_HUMAN	0.5	4.5	No
259	O75832	26S proteasome non-ATPase regulatory subunit 10	PSD10_HUMAN	0	4.5	No
260	O00233	26S proteasome non-ATPase regulatory subunit 9	PSMD9_HUMAN	0	4.5	No
261	O60711	Leupaxin	LPXN_HUMAN	0	4.5	No
262	P61289	Proteasome activator complex subunit 3	PSME3_HUMAN	0	4.5	Yes (Ewing, 2007)
263	P55735-2	Isoform 2 of Protein SEC13 homolog	SEC13_HUMAN	0	4.5	Yes (Ewing, 2007)
264	Q8IYS1	Peptidase M20 domain-containing protein 2	P20D2_HUMAN	0	4.5	No
265	Q8IUF8-4	Isoform 4 of Bifunctional lysine-specific demethylase and histid	MINA_HUMAN	0	4.5	No
266	Q9UHD8-7	Isoform 7 of Septin-9	SEPT9_HUMAN	0	4.5	No
267	Q9ULX6	A-kinase anchor protein 8-like	AKP8L_HUMAN	0	4.5	No
268	Q8N3U4	Cohesin subunit SA-2	STAG2_HUMAN	0	4.5	No
269	P59045-3	Isoform 3 of NACHT, LRR and PYD domains-containing protein	NAL11_HUMAN	0	4.5	No
270	O75330-4	Isoform 4 of Hyaluronan mediated motility receptor	HMMR_HUMAN	0	4.5	No
271	Q6RFH5	WD repeat-containing protein 74	WDR74_HUMAN	0.5	4	No
272	O75569-3	Isoform 3 of Interferon-inducible double stranded RNA-depende	PRKRA_HUMAN	0.5	4	No
273	Q9Y5Y2	Cytosolic Fe-S cluster assembly factor NUBP2	NUBP2_HUMAN	0.5	4	No
274	Q9Y3C1	Nucleolar protein 16	NOP16_HUMAN	0.5	4	No
275	Q8NEJ9-2	Isoform 2 of Neuroguidin	NGDN_HUMAN	0.5	4	No
276	Q92785	Zinc finger protein ubi-d4	REQU_HUMAN	0.5	4	No
277	Q9GZP4-2	Isoform 2 of PITH domain-containing protein 1	PITH1_HUMAN	0	4	No
278	P61758	Prefoldin subunit 3	PFD3_HUMAN	0	4	No
279	Q9UBV8	Peflin	PEF1_HUMAN	0	4	No
280	P68400	Casein kinase II subunit alpha	CSK21_HUMAN	0	4	No
281	Q9H501	ESF1 homolog	ESF1_HUMAN	0	4	No
282	Q7Z2W4	Zinc finger CCCH-type antiviral protein 1	ZCCHV_HUMAN	0	4	No
283	Q15628	Tumor necrosis factor receptor type 1-associated DEATH doma	TRADD_HUMAN	0.5	3.5	No
284	Q86YT6	E3 ubiquitin-protein ligase MIB1	MIB1_HUMAN	0.5	3.5	No
285	P25787	Proteasome subunit alpha type-2	PSA2_HUMAN	0	3.5	No
286	P09525	Annexin A4	ANXA4_HUMAN	0	3.5	No
287	P40925	Malate dehydrogenase, cytoplasmic	MDHC_HUMAN	0	3.5	No
288	Q16740	Putative ATP-dependent Clp protease proteolytic subunit, mitoc	CLPP_HUMAN	0	3.5	No
289	O95861	3'(2'),5'-bisphosphate nucleotidase 1	BPNT1_HUMAN	0	3.5	No
290	P23526-2	Isoform 2 of Adenosylhomocysteinase	SAHH_HUMAN	0	3.5	Yes (Ewing, 2007)
291	Q15054	DNA polymerase delta subunit 3	DPOD3_HUMAN	0	3.5	No
292	O15355	Protein phosphatase 1G	PPM1G_HUMAN	0	3.5	No
293	Q9UJU6	Drebrin-like protein	DBNL_HUMAN	0	3.5	No
294	Q8IWC1-2	Isoform 2 of MAP7 domain-containing protein 3	MA7D3_HUMAN	0	3.5	No
295	Q9H3P7	Golgi resident protein GCP60	GCP60_HUMAN	0	3.5	No
296	O00267-2	Isoform 2 of Transcription elongation factor SPT5	SPT5H_HUMAN	0	3.5	No
297	Q6ZU80	Centrosomal protein of 128 kDa	CE128_HUMAN	0	3.5	No
298	Q8IWJ2	GRIP and coiled-coil domain-containing protein 2	GCC2_HUMAN	0	3.5	No
299	Q9H9E3-3	Isoform 3 of Conserved oligomeric Golgi complex subunit 4	COG4_HUMAN	0	3.5	No
300	P36957	Dihydrolipoyllysine-residue succinyltransferase component of 2	ODO2_HUMAN	0.5	3	No
301	P41227-2	Isoform 2 of N-alpha-acetyltransferase 10	NAA10_HUMAN	0	3	Yes (Ewing, 2007)

302	Q96J01	THO complex subunit 3	THOC3_HUMAN	0	3	No
303	Q96P16	Regulation of nuclear pre-mRNA domain-containing protein 1A	RPR1A_HUMAN	0	3	No
304	P35520	Cystathionine beta-synthase	CBS_HUMAN	0	3	No
305	Q9UJU6-2	Isoform 2 of Drebrin-like protein	DBNL_HUMAN	0	3	No
306	P23458	Tyrosine-protein kinase JAK1	JAK1_HUMAN	0	3	No
307	Q9HA64	Ketosamine-3-kinase	KT3K_HUMAN	0	3	No
308	Q6PJT7-4	Isoform 4 of Zinc finger CCCH domain-containing protein 14	ZC3HE_HUMAN	0	3	No
309	Q86VM9	Zinc finger CCCH domain-containing protein 18	ZCH18_HUMAN	0	3	No
310	Q9Y2X9	Zinc finger protein 281	ZN281_HUMAN	0	3	No
311	Q9BUQ8	Probable ATP-dependent RNA helicase DDX23	DDX23_HUMAN	0	3	No
312	O14976	Cyclin-G-associated kinase	GAK_HUMAN	0	3	No
313	Q53H96	Pyrroline-5-carboxylate reductase 3	P5CR3_HUMAN	0	3	No
314	P52732	Kinesin-like protein KIF11	KIF11_HUMAN	0	3	No
315	Q96KA5-2	Isoform 2 of Cleft lip and palate transmembrane protein 1-like p	CLP1L_HUMAN	0	3	No
316	P55769	NHP2-like protein 1	NH2L1_HUMAN	0.5	2.5	No
317	Q15691	Microtubule-associated protein RP/EB family member 1	MARE1_HUMAN	0.5	2.5	Yes (Ewing, 2007)
318	Q15653-2	Isoform 2 of NF-kappa-B inhibitor beta	IKBB_HUMAN	0	2.5	Yes (Bouwmeester, 2004
319	Q9NUQ9	Protein FAM49B	FA49B_HUMAN	0	2.5	No
320	P62993	Growth factor receptor-bound protein 2	GRB2_HUMAN	0	2.5	Yes (Ewing, 2007)
321	Q96EY8	Cob(I)yrinic acid a,c-diamide adenosyltransferase, mitochondria	MMAB_HUMAN	0	2.5	No
322	P51553-2	Isoform 2 of Isocitrate dehydrogenase [NAD] subunit gamma, n	IDH3G_HUMAN	0	2.5	No
323	Q9BTE7	DCN1-like protein 5	DCNL5_HUMAN	0	2.5	No
324	Q16637-4	Isoform SMN-delta57 of Survival motor neuron protein	SMN_HUMAN	0	2.5	No
325	Q9H8H0	Nucleolar protein 11	NOL11_HUMAN	0	2.5	No
326	Q9BV38	WD repeat-containing protein 18	WDR18_HUMAN	0	2.5	No
327	P08758	Annexin A5	ANXA5_HUMAN	0	2.5	No
328	Q6JBY9	CapZ-interacting protein	CPZIP_HUMAN	0	2.5	No
329	Q9Y4R8	Telomere length regulation protein TEL2 homolog	TELO2_HUMAN	0	2.5	No
330	Q92540-2	Isoform 2 of Protein SMG7	SMG7_HUMAN	0	2.5	No
331	Q76FK4-4	Isoform 4 of Nucleolar protein 8	NOL8_HUMAN	0	2.5	No
332	Q9UJC3	Protein Hook homolog 1	HOOK1_HUMAN	0	2.5	No
333	P11802	Cyclin-dependent kinase 4	CDK4_HUMAN	0	2.5	Yes (Ewing, 2007)
334	Q00535-2	Isoform 2 of Cyclin-dependent kinase 5	CDK5_HUMAN	0	2.5	No
335	P04083	Annexin A1	ANXA1_HUMAN	0	2.5	Yes (Sigglekow, 2012)
336	P43487	Ran-specific GTPase-activating protein	RANG_HUMAN	0.5	2	Yes (Ewing, 2007)
337	P54920	Alpha-soluble NSF attachment protein	SNAA_HUMAN	0.5	2	No
338	Q6UXN9	WD repeat-containing protein 82	WDR82_HUMAN	0.5	2	No
339	Q14790-8	Isoform 8 of Caspase-8	CASP8_HUMAN	0.5	2	No
340	Q96T51	RUN and FYVE domain-containing protein 1	RUFY1_HUMAN	0.5	2	No
341	Q86Y82	Syntaxin-12	STX12_HUMAN	0.5	2	No
342	Q9NXW2	DnaJ homolog subfamily B member 12	DJB12_HUMAN	0.5	2	No
343	P16591-3	Isoform 3 of Tyrosine-protein kinase Fer	FER_HUMAN	0.5	2	No
344	Q9BVC4	Target of rapamycin complex subunit LST8	LST8_HUMAN	0.5	2	No
345	Q8IZP0-10	Isoform 10 of Abl interactor 1	ABI1_HUMAN	0	2	No

346	P48556	26S proteasome non-ATPase regulatory subunit 8	PSMD8_HUMAN	0	2	Yes (Ewing, 2007)
347	Q9H6Y2	WD repeat-containing protein 55	WDR55_HUMAN	0	2	No
348	Q9NQT4	Exosome complex component RRP46	EXOS5_HUMAN	0	2	No
349	Q15003	Condensin complex subunit 2	CND2_HUMAN	0	2	No
350	P38432	Coilin	COIL_HUMAN	0	2	No
351	Q86X83	COMM domain-containing protein 2	COMD2_HUMAN	0	2	No
352	Q6IQ49-2	Isoform 2 of Protein SDE2 homolog	SDE2_HUMAN	0	2	No
353	P42574	Caspase-3	CASP3_HUMAN	0	2	No
354	Q3KQU3-2	Isoform 2 of MAP7 domain-containing protein 1	MA7D1_HUMAN	0	2	No
355	P39687	Acidic leucine-rich nuclear phosphoprotein 32 family member A	AN32A_HUMAN	0	2	No
356	P78417-3	Isoform 3 of Glutathione S-transferase omega-1	GSTO1_HUMAN	0	2	No
357	O00273	DNA fragmentation factor subunit alpha	DFFA_HUMAN	0	2	Yes (Ewing, 2007)
358	Q7Z460-2	Isoform 2 of CLIP-associating protein 1	CLAP1_HUMAN	0	2	No
359	Q5JSZ5	Protein PRRC2B	PRC2B_HUMAN	0	2	No
360	O15118	Niemann-Pick C1 protein	NPC1_HUMAN	0	2	No
361	Q9NWH9	SAFB-like transcription modulator	SLTM_HUMAN	0	2	No
362	Q9Y263	Phospholipase A-2-activating protein	PLAP_HUMAN	0	2	No
363	P15927	Replication protein A 32 kDa subunit	RFA2_HUMAN	0	2	No
364	Q9Y697-2	Isoform Cytoplasmic of Cysteine desulfurase, mitochondrial	NFS1_HUMAN	0	2	No
365	Q9H3N1	Thioredoxin-related transmembrane protein 1	TMX1_HUMAN	0	2	Yes (Ewing, 2007)

The human MM cell line 8226 cells were transduced with pUB-hMCC-SBP-6xHis or pUB-FLAG-hMCC. Immunoprecipitates of hMCC-SBP-6xHis by streptavidin-sepharose beads from whole cell lysates and purified mitochondria of 8226 cells were analyzed by high resolution LC-MS/MS, respectively. Immunoprecipitates of FLAG-hMCC by streptavidin-sepharose beads were used as negative control in these experiments. LC-MS/MS data were searched against the human IPI and UniProt databases using the Mascot and Proteome Discoverer search engines. Protein assignments were considered highly confident using a stringent false discovery rate threshold of <1%, as estimated by reversed database searching, and requiring that ≥2 peptides per protein be unambiguously identified. Rough relative protein amounts were estimated using spectra counting values, and requiring that ≥2 of average spectra count difference between hMCC-SBP-6xHis and FLAG-hMCC (negative control) of two experiments. Proteins that were previously identified as MCC-interactors in CRCs or 293T cells are indicated. SBP: streptavidin binding peptide tag

References:

Ewing RM, Chu P, Elisma F, Li H, Taylor P, Climie S et al. Large-scale mapping of human protein-protein interactions by mass spectrometry. *Mol Syst Biol* 2007; **3**: 89.

Sigglekow ND, Pangon L, Brummer T, Molloy M, Hawkins NJ, Ward RL et al. Mutated in colorectal cancer protein modulates the NF-κB pathway. *Anticancer Res* 2012; **32**: 73-79.

Bouwmeester T, Bauch A, Ruffner H, Angrand PO, Bergamini G, Croughton K et al. A physical and functional map of the human TNF-α/NF-κB signal transduction pathway. *Nat Cell Biol* 2004; **6**: 97-105.
