# Supplementary material for: Mutated in colorectal cancer (MCC) is a novel oncogene in B lymphocytes
Source: J Hematol Oncol. 2014 Sep 9;7:56. doi: 10.1186/s13045-014-0056-6 (PMC4172902; doi:10.1186/s13045-014-0056-6)
Supplement: Additional file 3: Figure S1. — The MCC isoform 2 was identified as an MCC-interacting protein by affinity purification and LC-MS/MS. The two isoforms of MCC proteins differ at their extreme N-terminus due to alternative promoter usage. Both pUB-FLAG-hMCC and pUB-hMCC-SBP-6xHis are cloned from MCC isoform 1 (829 aa). (A) Schematic diagram of peptide sequences of MCC isoform 1 identified by LC-MS/MS. (B) Schematic diagram of peptide sequences of MCC isoform 2 (1019 aa) identified by LC-MS/MS. The peptide sequences detected by LC-MS/MS are highlighted in green color. The unique region of MCC isofrom 2 is marked with a dashed black box. [file 13045_2014_56_MOESM3_ESM.pdf]

Supplementary Figure 1

A MCC isoform 1: 82.63% coverage

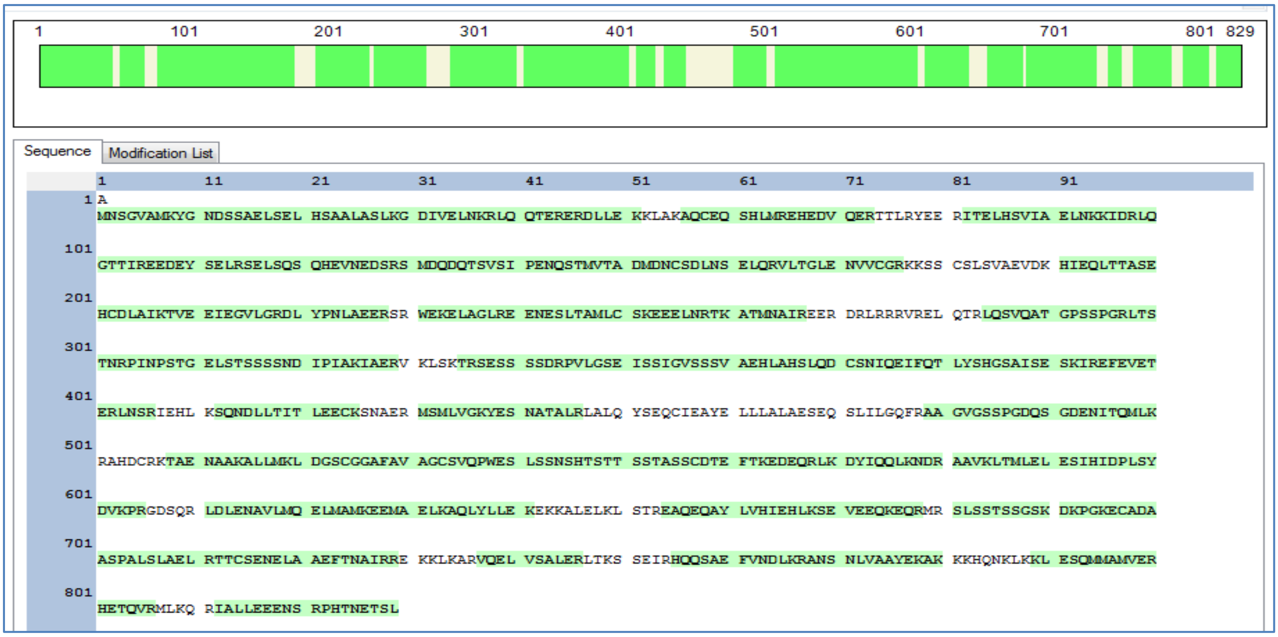

B MCC isoform 2: 74.29% coverage

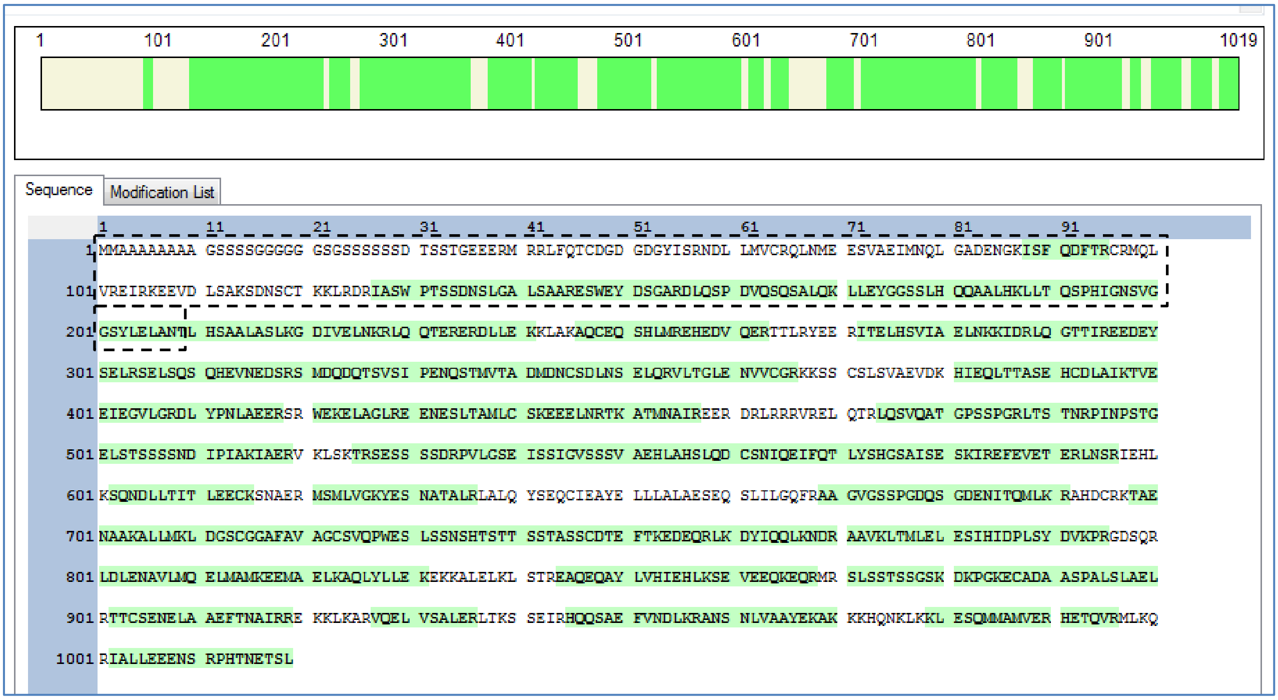

Supplementary Figure 1. The MCC isoform 2 was identified as an MCC-interacting protein by affinity purification and LC-MS/MS. The two isoforms of MCC proteins differ at their extreme N-terminus due to alternative promoter usage. Both pUB-FLAG-hMCC and pUB-hMCC-SBP-6xHis are cloned from MCC isoform 1 (829 aa). (**A**) Schematic diagram of peptide sequences of MCC isoform 1 identified by LC-MS/MS. (**B**) Schematic diagram of peptide sequences of MCC isoform 2 (1019 aa) identified by LC-MS/MS. The peptide sequences detected by LC-MS/MS are highlighted in green color. The unique region of MCC isofrom 2 is marked with a dashed black box.
